# Connecting the Dots: Bridging Microsamples and Conventional Blood Matrices in Metabolic Biomarker Analysis

**DOI:** 10.1002/ansa.70044

**Published:** 2025-09-14

**Authors:** Marlene Thaitumu, Vasiliki Gkanali, Georgios Theodoridis, Helen Gika

**Affiliations:** ^1^ Department of Medicine Aristotle University of Thessaloniki Thessaloniki Greece; ^2^ Biomic Center for Interdisciplinary Research and Innovation (CIRI‐AUTH) Thessaloniki Greece; ^3^ Department of Chemistry Aristotle University of Thessaloniki Thessaloniki Greece

**Keywords:** blood metabolites, blood microsampling, capillary blood, dried blood spots (DBSs), metabolomics, metabolic biomarker, microsampling devices, plasma, serum, volumetric absorptive microsampling (VAMS)

## Abstract

Bridging the gap between microsampling techniques and standard blood matrices presents a groundbreaking opportunity in metabolic biomarker analysis, offering minimally invasive, patient‐centric alternatives to traditional venipuncture. This review presents the current knowledge obtained from the comparison of biomarkers analysis in liquid blood, plasma or serum in parallel to blood microsamples by targeted and untargeted metabolic profiling assays. It aims to explore the analytical performance of these approaches compared to conventional blood collection, emphasizing its efficacy in the field. Key challenges such as haematocrit effect and validation studies which are necessary steps for standardization and widespread clinical adoption are pointed out through examples in different applications. This review underscores the critical steps to employ the full potential of microsampling technologies for their integration in metabolic biomarker discovery and clinical diagnostics.

## 1 Introduction

Blood microsampling (BµS) has revolutionized the field of bioanalysis by enabling the collection of small volumes of blood with minimal invasiveness. Through its ‘patient‐centric’ character, microsampling technologies are particularly useful in clinical research and have lately gained interest in the analysis of small endogenous metabolites that may serve as diagnostic markers.

Microsampling involves the collection of small volumes of capillary blood, usually <100 µL for dried microsamples and up to 1000 µL for liquid microsamples [1, 2]. The oldest form of microsampling was reportedly performed by Ivar Bang in 1913 who analysed glucose from a dried blood spot (DBS) [3]. DBS later became a gold standard for newborn screening after Guthrie and Susi used it for analysis of phenylalanine, a phenylketonuria (PKU) biomarker [4]. Since then, DBS has gained interest and usage in various applications, including therapeutic drug monitoring (TDM), toxicology, sports doping and forensics [5, 6, 7, 8, 9, 10, 11, 12].

BµS emergence in biomarker discovery and analysis is supported by the analytical technologies applied in this area such as liquid or gas chromatography mass spectrometry (LC–MS, GC–MS), which, thanks to their sensitivity, allow for the use of small sample volumes [13]. Besides this, there are certain advantages that are offered by BµS, including facilitation of sample collection in remote geographical regions or at home with no trained nurses and potentials for point‐of‐care diagnostics; convenience for phlebotomy‐averse populations (e.g., newborns, children and elderly) or chronically ill requiring frequent examinations; simpler sample transportation logistics (e.g., no refrigeration required), thus reduced shipping costs and storage; integration in automated analysis and enhanced stability of certain molecules [14, 15, 16, 17]. These advantages showcase BµS as an attractive alternative to venipuncture with high potentials in contributing to the revolutionization of human healthcare.

Despite the multiple advantages BµS presents over conventional blood matrices in bioanalysis, its implementation in the analysis of endogenous biomarkers of diseases has been slow and faces several limitations. For example, some biomarkers may be less stable [16, 17, 18] or unrecoverable from dried BµS, the small sample volumes limit the range (low abundant ones) of metabolites analysed, haematocrit (HCT) can lead to variable results, and most importantly, the levels of metabolites may differ between capillary blood (from finger‐prick or upper arm) and venous blood as the latter is composed of arterial, venous and interstitial fluid. Additionally, regulatory measures for BµS are still evolving, and differences in procedures, including sampling, storage and extraction, may hamper comparability of results. Therefore, validation studies and enough data for bridging results between conventional matrices (liquid venous blood, plasma and serum) and BµS (liquid and dried) are required in order to confirm the accuracy, precision and feasibility of BµS for routine bioanalysis of small molecules.

There are several works reviewing applications of various BµS devices in bioanalysis over the last 8 years. DBS, capillary microsampling (<32 µL blood collected in a glass capillary) and Mitra's applications in the omics, pre‐clinical studies, diagnosis, TDM and forensic toxicology were compared in 2017 [18]. Then, Lei et al. in 2019 presented different blood and skin µS techniques, their significance in the clinical field and their social impact [19]. Thangavelu et al. [1] gave an overview of the technological advancements and applications of BµS in 2022, whereas Couacault et al. recently [20] focused on analytical methods, applications and perspectives of dried BµS. In addition, there are reviews based on either only one BµS device, for example, Mitra and its application in endogenous metabolites [21, 22] on analytical workflows [23], on TDM and pharmacokinetic studies [14, 24], on DBS only [25], on automation of DBS samples analysis [26] and on advances of various BµSs for TDM [27].

Although all these reviews have highlighted the impact of BµS in various scientific fields, none have touched on bridging validation studies between BµS and conventional matrices for various biomolecules with the aim of supporting the potential of BµS approaches in the analysis of small biomolecules and metabolic markers.

In this review, we aim to provide an insight on the current knowledge of the potential of microsampling devices in the bioanalysis for endogenous metabolites. The review has a special focus on the latest information on results obtained from comparative studies of dried BµS and liquid BµS devices with conventional blood specimens (serum, plasma or whole blood). Thus, herein works on the analysis of endogenous metabolites by targeted assays and untargeted metabolomics methodologies using mass spectrometry (MS) techniques or enzymatic assays in at least one type of BµS in parallel with blood specimens are encompassed with the goal to present data bridging the results of the two blood sample types.

To the best of our knowledge, this is the first review concentrating on the direct comparison of measurements of endogenous metabolites in liquid and dried BµS versus conventional blood matrices.

## 2 Methodology

The literature survey was performed with the aim of finding articles presenting data on the analysis of metabolic biomarkers in both conventional blood specimens (liquid venous blood, plasma or serum) and dried blood or liquid microsample from the same individuals either by targeted or untargeted analysis. The literature search was performed in ‘PUBMED’ using the keywords: ‘DBS metabolomics blood’, ‘DBS metabolomics plasma’, ‘biomarkers DBS plasma’, ‘biomarkers microsampling plasma’, ‘DBS untargeted metabolomics plasma’ and ‘DBS targeted metabolomics plasma’. Searches were also performed using the same phrases but substituting DBS with other BµS devices. Further searches for publications were done on individual microsampling vendors websites. The search date range was limited from January 2021 to January 2025. However, for untargeted metabolomics, works were limited; therefore, articles from 2017 to 2025 were included.

**FIGURE 1:**
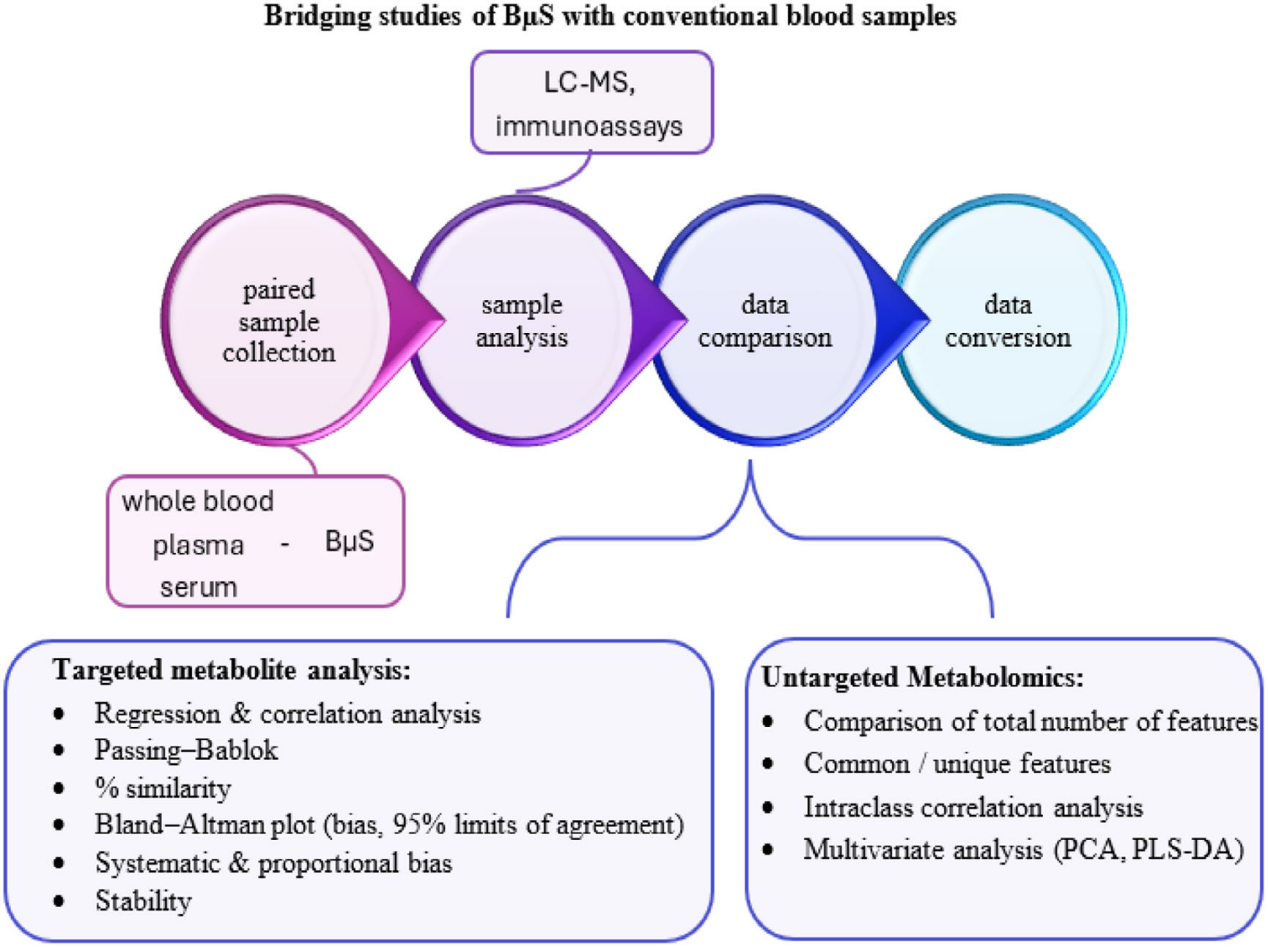
Schematic depiction of the comparison process from sample collection to data analysis in targeted biomarker analysis and untargeted metabolomics. BµS, blood microsampling/microsample; LC–MS, liquid chromatography–mass spectrometry.

The articles retrieved were considered further only when they included data on comparative studies between BµS and conventional blood specimens. It was thus a prerequisite to present concentration data or criteria of agreement from the analysis of both human blood and BµS. For untargeted metabolomics studies, other qualitative data were taken into account. Only works that involved analysis of endogenous metabolites of low molecular weight (<1000) were considered. It should be noted that a number of manuscripts describing analysis of only BµS were excluded. On the basis of that criteria, a total of 18 manuscripts on targeted biomarker analysis and eight on untargeted metabolomics were collected. Given the scarcity of comparative studies in untargeted metabolomics, the corresponding section is proportionally shorter in this manuscript. A schematic depiction of the comparison process from sample collection to data analysis in the studies presented here can be seen in Figure 1.

## 3 BµS Approaches and Devices

Different types of microsampling collectors exist, from the older form of DBS to more novel technologies offering further advantages. As DBS faces challenges, including HCT effects, which can lead to different blood spot sizes, inaccurate blood spot volumes and sample inhomogeneity, newer volumetric microsampling devices have been developed with the aim to mitigate these issues, therefore allowing the collection of a precise, fixed volume of blood [14, 23, 24, 28, 29]. There is the volumetric absorptive microsampling (VAMS) device Mitra, microfluidics collection devices, including the Hemaxis DB 10 and Capitainer B, and the vacuum‐assisted collection device, TASSO‐M20 [14, 30, 31, 32, 33, 34].

In addition to dried BµS, liquid BµS devices have emerged as viable collection techniques for small molecule analysis. Similar to the others, they collect specific but larger volumes of blood. Some of the devices include Touch Activated Phlebotomy (TAP II), TASSO+, TASSO + serum separator tube (SST), Onflow Serum Gel (OSG) and BD Microtainers [2, 32, 33, 34, 35]. Finally, there are devices which use microchannels to collect and separate small amounts of blood. This technology is still emerging, with higher costs compared to traditional methods.

In this review, the studies described have used the following devices and include both absorptive and those collecting liquid capillary blood:

1. **DBS cards**: Capillary blood drops are collected on Whatman or other cards made of cellulose fibre [18].
2. **BD microtainers (BDµM)**: Collects capillary blood from a finger prick. The collected blood is not volumetrically accurate [2].
3. **Quantitative dried blood spot (qDBS), Capitainer B**: Capillary blood is collected on an inlet port which fills a connecting capillary channel with a predefined volume. A thin film on the outlet dissolves allowing blood in the capillary to empty into a pre‐perforated paper disc [30, 36].
4. **VAMS, Mitra**: Specific volumes of capillary blood, for example, 10, 20 or 50 µL, are collected on adsorbent polymeric tips [21].
5. **TASSO‐M20**: Capillary blood is collected from the upper arm by a vacuum system which draws specific volumes of blood into a DBS collection device [32, 33, 34].
6. **The Hemaxis DB 10 (HDB)**: Specific volumes of capillary blood are collected in a foldable cassette with four microfluidic channels on one side and an absorbent filter paper on the other [31].
7. **TAP II**: It consists of a plunger connected to bladeless microneedles and a microcapillary tube for blood collection of up to 500 µL from the upper arm in seconds [2, 37].
8. **TASSO+**: It is composed of a lancet and a micro‐collection tube at the bottom which collects capillary blood from the upper arm. A vacuum system draws 200–600 µL blood into the tube. TASSO + SST has an SST connected for serum separation [32, 33, 34].
9. **OSG loop**: The OSG accesses the blood just below the skin and can collect 1.5 mL of capillary blood in 2–6 min from the upper arm into an attached micro‐SST tube [35, 38].

## 4 Metabolic Biomarkers Analysis

Blood‐based metabolic biomarkers, both classical and novel, play pivotal roles in the early diagnosis, prognosis and monitoring of a wide spectrum of diseases. Classical biomarkers include well‐established analytes such as glucose, cholesterol, creatinine, urea, lactate and others which are routinely measured in clinical laboratories to assess metabolic function and detect several disorders [39, 40]. Moreover, by utilizing high‐throughput analytical platforms such as MS, the quantification of endogenous low‐molecular‐weight metabolites that are involved in subtle biochemical alterations linked to disease processes is enabled leading to novel biomarkers analysis. Longitudinal monitoring of biomarkers providing insights into disease progression, treatment efficacy and integration of metabolic biomarkers into clinical workflows could be greatly supported by patient‐centric sampling approaches such as BµS. This requires thorough extensive clinical validation to demonstrate that results are comparable to traditional venipuncture methods and to build confidence among healthcare professionals and patients of their efficacy and reliability.

Several studies have been performed on the analysis of various biomarkers by ΒµS. Studies reporting comparisons of blood samples collected by BµS against conventional blood matrices in the targeted analysis of endogenous metabolites are presented here (see Table 1). Devices used in these studies involve Capitainer, Mitra, hemaPEN, DBS, TAP II, TASSO+, TASSO + SST, HDB, TASSO‐M20, OSG and BDµM; their findings based on the measurements are herein described.

**TABLE 1:** Studies measuring metabolites in parallel by targeted assays in blood microsamples (BµS) and liquid blood, plasma or serum.

Metabolites measured	Matrix/Microsampling device	Analytical technique	Outcome	Analytical data	Reference
BIL, BUN, cholesterol, CO_2_, creatinine, glucose, HDL and TRIG	TAP II and BDµM vs. serum	Enzymatic assay	The study demonstrated the viability of TAP II and BDµM as an option for patient‐centric sampling in clinical chemistry panel analysis	Table S1	[2]
Glucose, creatinine, phosphorous	TASSO + SST vs. serum	Enzymatic assay	The TASSO + SST centrifuged samples had substantial correlations for creatinine, glucose and phosphorus when compared to serum, whereas TASSO + SST liquid blood had almost perfect correlation for creatinine, substantial for phosphorus, and poor for glucose when compared to serum	Table S1	[33]
LDH and creatinine	OSG vs. serum	Enzymatic assay	The results showed that the OSG is feasible for collection and analysis of creatinine and lactulose dehydrogenase; despite its negative bias, the results were within the acceptance criteria	Table S1	[35]
Creatinine	Venous Capitainer vs. plasma	LC–MS/MS	Venous blood on Capitainer showed accuracy in creatinine analysis	Table S2	[41]
Creatinine	Venous Capitainer vs. venous Mitra vs. plasma	FIA‐MS/MS and enzymatic assay	The study concluded that although Capitainer and Mitra BµS are feasible for measurement of creatinine, correction and conversion is required in order to compare to plasma. Additionally, Capitainer had the highest consistency and analytical quality when patients self‐sampled	Table S2	[42]
Creatinine	**Capillary** DBS (supervised and unsupervised), venous DBS and plasma	LC–MS/MS and enzymatic assay	In all cases a strong correlation of creatinine concentrations was found, therefore, suggesting that creatinine can be accurately and precisely measured in capillary DBS, providing a suitable sampling option for patients with chronic kidney disease	Table S2	[43]
Phenylalanine	**Capillary** Capitainer and plasma	LC–MS/MS	The results indicated that Capitainer could ensure higher sample quality especially in venepuncture‐averse populations for example newborns and children	Table S2	[44]
Amino acids, its derivatives and carnitines	Venous DBS and plasma	FIA‐MS/MS	The results showed that, unlike plasma, venous DBS is able to provide cellular metabolism information in the red blood cells including acylcarnitine profiles with higher sensitivity	Table S2	[45]
Globotriaosylsphingosine (lyso‐Gb3) and analogues	**Capillary** DBS, capillary Capitainer, venous DBS, venous Capitainer and plasma	LC–MS/MS	Results showed that Capitainer and DBS are feasible for screening, follow‐up and monitoring of patients with Fabry disease	Table S2	[46]
25OHD3, 25OHD2 and 3‐epi‐25OHD3	Venous DBS, **capillary** DBS and plasma	LC–MS/MS	The results of this study indicated that a linking strategy is required between plasma and DBS data for accurate results	Table S2	[47]
25OHD3 and 25OHD2	Venous DBS, **capillary** DBS and plasma	LC–MS/MS	The study showed that 25OHD and its metabolite can be accurately measured in DBS as compared to plasma	Table S2	[48]
Vitamin A	Venous DBS, **capillary** DBS and plasma	LC–MS/MS	In summary, the accurate and precise measurements of retinol in DBS show its potential for future routine use	Table S2	[49]
Testosterone, epitestosterone, A4, P, 17𝞪OHP, DHEA, DHT, corticosterone, cortisol, deoxycorticosterone and 11‐deoxycortisol	Venous DBS, serum and plasma	LC–MS/MS	Overall venous DBS steroids concentrations were between those in serum and plasma, correlating with either depending on the analyte. The presence of anticoagulants in venous DBS, however, could be a source of ion suppression. TES and A4 venous DBS concentrations had the highest agreements with serum and plasma	Table S2	[50]
25OHD3 and testosterone	Venous DBS and plasma	LC–MS/MS	In summary, the study showed that standardization of DBS results may be required for better interpretations of DBS results using plasma references or cut‐off values	Table S2	[51]
NAD+ and NMN	Liquid blood and venous DBS	LC–MS/MS	The study showed that NAD+ and NMN can be stored with high stability in DBS, particularly DMPK‐B paving a way for accurate analysis and aging‐related diseases research	Table S2	[52]
Uracil	TASSO + SST and plasma	LC–MS/MS	The study showed that TASSO + SST samples can be used for accurate measurements of uracil as compared to plasma	Table S3	[32]
PEth	TASSO‐M20 and liquid blood	LC–MS/MS	The PEth concentrations between TASSO‐M20 and liquid blood were highly correlated, importantly at low concentration levels (<200 ng/mL)	Table S3	[34]
PEth	Venous DBS, venous Capitainer, venous Mitra, venous HDB, liquid venous blood	LC–MS/MS	The results confirmed post‐sampling formation of PEth in liquid blood samples which contain ethanol. The same result was observed in the other BµS devices excluding DBS which makes it suitable for PEth analysis	Table S3	[31]

Abbreviations: BIL, bilirubin; BUN, blood urea nitrogen; DBS, dried blood spot; DHEA, dehydroepiandrosterone; DHT, dihydrotestosterone; HDL, high density lipoprotein; LC–MS, liquid chromatography–mass spectrometry; LDH, lactulose dehydrogenase; NAD, nicotinamide adenine dinucleotide; NMN, nicotinamide mononucleotide; OSG, onflow serum gel; PEth, phosphatidylethanol; SST, serum separator tube; TAP II, Touch Activated Phlebotomy; TES, testosterone; TRIG, triglycerides.

### 4.1 Evaluation of Liquid BµS Devices as Feasible Collection Techniques in Clinical Chemistry Panels by Immunoassays

Collier et al. [2] compared two serum micro‐collection devices (TAP II and BDµM) used for capillary blood collection with serum obtained by venipuncture to validate BµS in blood chemistry and lipid panels, including bilirubin (BIL), blood urea nitrogen (BUN), cholesterol, carbon dioxide, creatinine, glucose, high‐density lipoprotein (HDL) cholesterol and triglycerides (TRIG). The experiments included a comparison of the chemistry panels in four types of samples: TAP II professionally collected, TAP II self‐collected, BDµM and serum collected from 43 subjects. In addition, a comparison of haemolysis index (HI) (sample quality and haemoglobin concentration) of samples from the four collection methods was performed. All analyses were performed by an enzymatic assay [2].

BIL, BUN, HDL, cholesterol and TRIG measurements in the devices had acceptable correlation (*R* ^2^ > 0.95) with those in serum. The mean biases and slopes (0.9–1.1) were within clinical laboratory improvement amendments (CLIA) limits. The agreement was >90% for all the metabolites, except for TRIG. The correlation for creatinine between all matrices was *R* ^2^ > 0.93. The slopes of the scatter plots (0.868–0.987) and mean biases (−8.1% to −10.9%) indicated lower concentrations of creatinine in the TAP II and BDµM collections compared to venous serum. Carbon dioxide (CO_2_) and glucose had poor correlations (*R* ^2^ < 0.67). Mean % bias for CO_2_ was −14%, whereas the bias for glucose was within CLIA limits despite a poor <55% agreement.

HI measurements are important for enzymatic assays because they can compromise measurements. Increased HI values are directly proportional to higher metabolite/lipids measurements. Serum had statistically lower HI (*p* < 0.001) in comparison to the other collection methods. Regardless of the variation in HI values, the haemolysis levels for all the collections were acceptable for most of the targets.

Venous serum and capillary serum (collected by TAP II and BDµM) showed acceptable correlations for analytes with >10% coefficient of variation (CV) (group biological variation). However, the correlation results could be positively impacted by using study samples containing wider concentration ranges. Nonetheless, the study demonstrated the viability of TAP II and BDµM as an option for patient‐centric sampling in clinical chemistry panel analysis except for glucose and CO_2_.

In another study by Wickremsinhe et al., serum obtained by venipuncture was compared with capillary blood collected using TASSO + SST from the upper arm [33]. The study was conducted on 36 patients (24 males and 12 females; ages 27–86) suspected of having abnormal liver function which may be caused by liver cirrhosis, viral hepatitis, metabolic and autoimmune diseases or cancer [53, 54, 55]. Such conditions require frequent monitoring of the disease biomarkers to ensure proper response to treatment and prevention of fatal outcomes, making BµS a beneficial approach for patients’ long‐term health.

Serum samples were collected by venipuncture and capillary blood samples using the TASSO + SST by a professional at a clinic (*n* = 2 for each collection type per study subject). Two types of TASSO+ samples were collected: TASSO + SST centrifuged (for serum collection) and regular TASSO + SST (for capillary liquid blood). Samples were analysed using an enzymatic assay for creatinine, glucose and phosphorus. Acceptance criteria for the agreement of results were adopted from McBride where concordance correlation coefficient (CCC) >0.99 is almost perfect, 0.95–0.99 is substantial, 0.90–0.95 is moderate, and <0.90 is poor [56, 57].

The TASSO + SST centrifuged versus serum CCC was above 0.95 for creatinine, glucose and phosphorus. The Deming regression slopes were as follows: 1.1001 for creatine, 1.0236 for glucose and 1.0788 for phosphorus. All slopes were within the 95% confidence interval (CI). The TASSO + SST liquid blood versus serum CCC was >0.99 for creatinine, >0.95 for phosphorus and <0.90 for glucose. The Deming regression slopes were as follows: 1.0138 for creatinine, 0.9883 for glucose and 1.0442 for phosphorus with all slopes being within the 95% CI.

In summary, the TASSO + SST centrifuged samples had substantial correlations for creatinine, glucose and phosphorus when compared to serum, whereas TASSO + SST liquid capillary blood had almost perfect correlation for creatinine, substantial for phosphorus and poor for glucose when compared to serum. However, these results were based on a small patient group size, and therefore, a larger demographic group with diverse reference ranges would be required for more accurate conclusions. In addition, different storage conditions were applied which deserves further investigation.

In another study, OSG was evaluated as a viable device for measurement of two metabolites, creatinine and lactulose dehydrogenase (LDH) from the chemistry blood panel using an enzymatic assay [35]. One hundred healthy participants were recruited for sample collection at a clinic by a professional. Two OSG samples were collected (from each arm), and one venous blood sample was drawn for serum collection. Following McBride [57] criteria, moderate agreement was found between the OSG and the serum creatinine (CCC at 0.912, within 95% CI), whereas the Pearson precision was 0.972. Analysis on a Bland–Altman plot showed that the OSG creatinine levels were lower than those in the serum. On the Bland–Altman, the mean bias was −5.633 µmol/L, mean similarity 96%, similarity standard deviation (SD) 2.7% and similarity CV 2.8%. There was mild to insignificant haemolysis (due to OSG collection time) on creatinine concentration.

For LDH analysis, agreement between the OSG and the serum was poor, with a CCC of 0.673 (within 95% CI) and a Pearson precision of 0.724. A Bland–Altman plot showed that the OSG LDH levels were lower when compared to serum. On the Bland–Altman, the mean bias was 14.707 µmol/L, mean similarity 104%, % similarity SD 7.0% and similarity CV 6.7%.

The results showed that the OSG is feasible for collection and analysis of creatinine and LDH. Despite its negative bias, the results were within the acceptance criteria. Considering that 14.1% of the participants did not prefer venipuncture collection compared to 3% who disliked OSG, its applicability is clear. However, even larger studies are required with participants collecting their own samples for more thorough validations in order to integrate OSG in routine clinical analysis which will improve workflows and reduce clinic visits.

### 4.2 Evaluation of BµS Devices in the Analysis of Metabolic Markers of Diseases by Mass Spectrometry

#### 4.2.1 Amino Acids, Their Derivatives and Carnitines

Some of the advantages of ΒµS, including the ease of use in frequent sample collection [14], find great application in chronically ill patients that need timely follow‐up often for monitoring of biomarkers. An example is kidney transplant patients that are subjected to frequent monitoring for kidney function and detection of nephrotoxicity [58, 59]. Creatinine is an important biomarker of kidney function; thus, BµS have been used a lot in the analysis of creatine in blood.

Creatinine levels and HCT effects were compared in venous blood collected on Capitainer and plasma samples [41]. Leftover venous blood from patients (*n* = 131) receiving immunosuppressant therapy was used for the study. Creatinine‐d3 was used as a surrogate analyte in calibrators and QCs. A Passing–Bablok regression analysis of creatinine concentrations between plasma and Capitainer was first performed to determine the need for a correction factor. The 95% CI of the slope indicated the absence of a proportional difference, but the intercept indicated presence of a systematic error (did not include zero). As such, a Passing–Bablok regression equation (*y* = 5.139 + 0.963*x*) was used to correct Capitainer creatinine concentrations to the measurement procedure targets.

To assess HCT, a % difference plot of (Capitainer ‐ plasma)/plasma against % HCT was used. Linear regression of the % difference plot showed the *R* ^2^ to be −0.08 and slope −0.124 (95% CI: −0.848 to 0.1374). The HCT effect was not significant because the 95% CI of the slope included zero. Agreement between the venous Capitainer samples and plasma was 90% (within limits).

As no assays exist for creatinine measurement in whole blood (routinely plasma or serum is used), the concentration ratio between Capitainer and plasma samples was determined and found to be 0.85 in 131 paired samples. Capitainer creatinine concentrations were corrected as pointed out in the previous paragraph and then divided using the 0.85 conversion factor. The corrected and converted Capitainer creatinine concentrations and plasma concentrations were analysed on a Bland–Altman plot showing a mean % bias of −0.8 (95% CI: −2.92% to 1.32%). Using a Passing–Bablok regression, a 1.014 slope was acquired.

The approach used can be applied to accurately measure creatinine in dried whole blood; however, further bridging studies are required for capillary blood collected from fingertip or upper arm to showcase its applicability in a clinical setting.

In another study, the focus was on the validation of metabolites measurement, including creatinine sampled on two BµS devices, Capitainer and Mitra, against the standard plasma measurements [42]. Analysis of creatinine in the BµS was done using flow injection‐MS/MS and in plasma using an enzymatic‐colourimetric assay.

For the preliminary in vitro study, venous blood from 26 kidney transplant recipients was sampled on the two BµS devices in parallel to plasma collection. The linear regression indicated strong agreement of the measurements: between Capitainer and plasma, *R* ^2^ was 0.994 (slope 0.75), and between Mitra and plasma, *R* ^2^ was 0.991 (slope 0.91). The % difference between plasma and Capitainer was −12.9% ± 13.2%, and between plasma and Mitra 2.1% ± 13.5%. For both BµS devices, there was an increasing positive bias as creatinine concentrations decreased. Therefore, equations (Capitainer: *x*_estimated_ = (*y *− 5)/0.75 and Mitra: *x*_estimated_ = (*y* − 4.4)/0.91) from the regression analysis were used as correction algorithms of creatinine measurement in the BµS devices.

In a second study, capillary blood collected on Capitainer and Mitra samples was compared with plasma. Creatinine correction algorithms derived from an in vitro study as previously described were applied to achieve a median bias equal to zero. The mean differences between Capitainer and plasma were 11%–13% and between Mitra and plasma 2.8%–4.6%. The proportions which were within ±15% difference were 68%–76% for Capitainer and 79%–92% for Mitra.

The study concluded that although Capitainer and Mitra BµS are feasible for measurement of creatinine, correction and conversion are required in order to compare to plasma. Additionally, Capitainer had the highest consistency and analytical quality when patients’ self‐sampled.

Comparison of creatinine concentrations in plasma, capillary DBS and venous DBS was also the subject of another study [43]. Plasma and DBS samples were collected from 173 study participants. From these, 113 samples were collected from the finger on DBS by a professional (controlled collection) and 30 samples from the finger by the participants (unsupervised collection). The remaining 30 samples were prepared by pipetting venous blood onto DBS cards. Plasma samples were analysed using an enzymatic Roche assay and DBS samples using an LC–MS/MS method.

**FIGURE 2:**
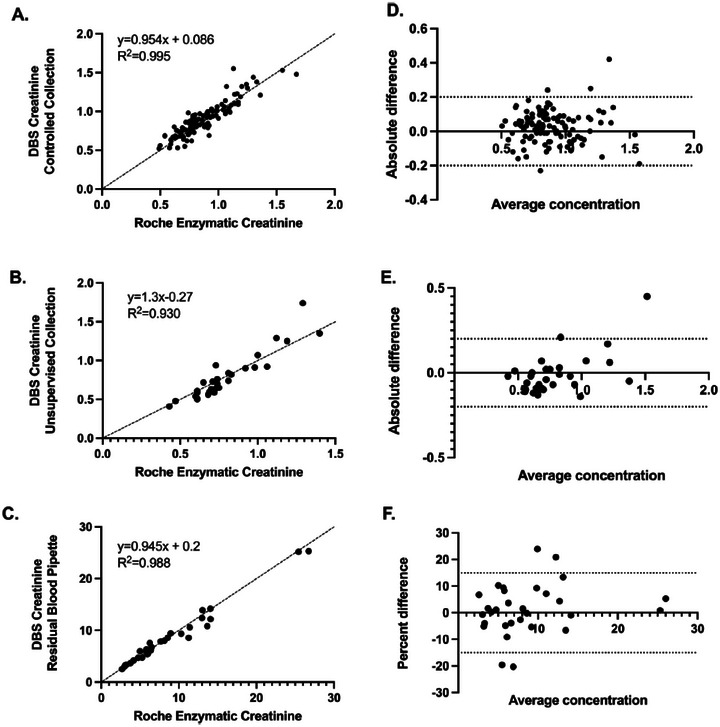
Regression and Bland–Altman plots showing creatinine concentration comparisons between plasma and DBS (capillary and venous) samples analysed using enzymatic and LC–MS/MS assays, respectively: (A) regression plot between controlled collected capillary DBS and plasma, (B) regression plot between unsupervised collected capillary DBS and plasma, (C) regression plot between venous DBS and plasma, (D) difference plot for controlled collected capillary DBS and plasma, (E) difference plot for unsupervised collected capillary DBS and plasma and (F) difference plot for venous DBS and plasma. DBS, dried blood spot. *Source*: Torres et al. [43].

The average bias between the three matrices was −0.04 mg/dL (−1.4%). In the regression analysis between the supervised capillary DBS and plasma, the *R* ^2^ was 0.9995, slope was 0.954, and intercept was 0.086. In the unsupervised capillary DBS versus plasma regression, *R* ^2^ was 0.930, the slope was 1.3, and the intercept was 0.27. In the venous DBS versus plasma regression, *R* ^2^ was 0.988, the slope was 0.945, and the intercept 0.2. The regression and Bland–Altman plots showing creatinine concentration comparisons between plasma and DBS (capillary and venous) samples analysed using enzymatic and LC–MS/MS assays, respectively, can be seen in Figure 2.

In all cases, a strong correlation of creatinine concentrations was found, suggesting that creatinine can be accurately and precisely measured in capillary DBS, providing a suitable sampling option for patients with chronic kidney disease.

Phenylalanine (Phe) levels in blood are critical for the diagnosis of the inherited disorder PKU which causes Phe to build up in the body. PKU can affect brain development if left untreated, and as such, frequent monitoring is important. For these reasons, BµS technologies could be proved beneficial for these patients. Carling et al. [44] investigated two ΒµS approaches for Phe monitoring. The study compared Phe concentrations of 51 PKU patients in plasma and Capitainer samples collected by finger prick. Samples were analysed using LC–MS/MS [44].

The Passing–Bablok regression results between plasma and Capitainer showed excellent agreement with *R* ^2^ 0.992, slope 0.8909 (95% CI: 0.8598–0.9130) and intercept −3.764 (95% CI: −17.31 to 5.042). The slope 95% CI did not include 1 indicating a proportional difference. A Cusum test (*p* = 0.9889) showed no significant deviation from linearity, and the RSD was 45.5 (95% CI: −89.2 to 89.2). The Bland–Altman mean bias was −13.3% (95% limits of agreement −29.03% to 2.44%).

The results indicated that Capitainer can ensure high sample quality especially in venepuncture‐averse populations, for example, newborns and children. The results have also set the basis for evaluation of volumetric absorptive devices in the analysis of other metabolic disorders such as tyrosinemia type 1, maple syrup urine disease and classical homocystinuria.

In another study, amino acids, their derivatives and carnitines measurements were compared in cancer cachexia patients versus healthy controls using venous blood collected on DBS and plasma [45]. Cancer cachexia is characterized by weight loss due to wastage of skeletal muscle mass and/or adipose tissue degradation in 30%–90% of patients usually in advanced stages of the disease [60, 61]. Analysis was conducted using FIA‐MS.

In the first study, venous DBS and plasma samples from 36 healthy controls were analysed and compared in order to determine the correlation between the two matrices. The Spearman *ρ* correlations were analysed on the basis of Burkhardt et al. guidelines [62]. They showed that proline and alanine had very strong correlations (*ρ* ≥ 0.8); aminobutyric acid, phenylalanine, threonine, carnitine free, alanine/acetylcarnitine, decanoyl carnitine, glycine and citrulline had strong correlation (*ρ* 0.6–0.79), whereas leucine/isoleucine, tyrosine, sarcosine, methyl histidine, butyrylcarnitine, arginine, acetylcarnitine, arginine/citrulline, serine, glutarylcarnitine/lysine, serine, glutarylcarnitine/lysine, hydroxyproline and pipecolic acid had moderate correlation (*ρ* 0.4–0.59).

In a second experiment, these metabolite levels were compared in cancer patients and controls in the same matrices. Arginine, citrulline and histidine were significantly low in both matrices. Asparagine, glutamine, methyl‐histidine, methionine, ornithine, serine, threonine and the ratio of leucine/isoleucine were significantly low in venous DBS but not in plasma. On the contrary, ornithine levels were significantly higher in plasma but not in venous DBS. In the acylcarnitines analysis, there was no clear pattern when comparing the two matrices in cancer patients and controls. Hexanoylcarnitine was significantly decreased in venous DBS and hexadecanoyl carnitine and octenoylcarnitine in plasma. On the other hand, free carnitine was significantly increased in plasma. Differences in carnitine ratios were only observed in venous DBS indicating their significant presence in the blood cells. As could be expected, and depending on the biomolecule structure, metabolite distribution between blood cells and plasma varies. Taking into account that different metabolic states such as disease conditions contribute to dynamic shifts of these equilibriums, it is clear that comparisons of biomolecules levels are even more important in plasma and whole blood in such cases.

The results overall showed that, unlike plasma, venous DBS is able to provide cellular metabolism information in the red blood cells, including acylcarnitine profiles with higher sensitivity.

#### 4.2.2 Lipid Species

In a recent study, Boutin et al. worked on the validation of Fabry disease (FB) biomarkers analysis in DBS. FB is a type of lysosomal storage disorder that causes mutations and impairment of 𝞪‐galactosidase and leads to accumulation of intracellular undegraded glycosphingolipids, including globotriaosylceramide (GB3), globotriaosylsphingosine (lyso‐Gb3) and galabiosylceramide (Ga2), among other analogues [46]. Clinical impacts of FB in children and adolescents include neuropathic pain, proteinuria, fatigue, gastrointestinal problems, impaired renal function and cardiomyopathy; it can lead to stroke in adults [63, 64].

For this study, venous DBS, venous Capitainer, capillary DBS and Capitainer were compared to plasma. Measurement of globotriaosylsphingosine (lyso‐Gb3) and its analogues in the four matrices was performed using LC–MS/MS.

As a first step, the normal reference values from 12 healthy controls were established for lyso‐Gb3 and its related analogues (lyso‐Gb3 −28 Da, lyso‐Gb3 −2 Da, lyso‐Gb3 +16 Da, lyso‐Gb3 +18 Da, lyso‐Gb3 +34 Da) in venous and capillary DBS and Capitainer. Deming regression analysis was conducted between plasma and capillary DBS (*n* = 32 patients). The 95% CI of the slope included one, indicating no proportional bias between lyso‐Gb3 and related analogues between the matrices. There was a systematic bias for lyso‐Gb3, lyso‐Gb3 −28 Da and lyso‐Gb3 −2 Da given the 95% CI of the intercept did not include zero. No systematic bias was found for the other three analogues. Bland–Altman analysis was also conducted between plasma and capillary DBS samples from the same 32 patients. The mean differences ± SD (95% limits of agreements) were as follows: 2.92 ± 1.33 nmol/L (0.31–5.53 nmol/L) for lyso‐Gb3 2.93; 0.17 ± 0.15 nmol/L (−0.13 to 0.47 nmol/L) for lyso‐Gb3 −28 Da; 0.19 ± 0.29 nmol/L (−0.37 to 0.76 nmol/L) for lyso‐Gb3 −2 Da; 0.01 ± 0.10 nmol/L (−0.19 to 0.22 nmol/L) for lyso‐Gb3 +16 Da; −0.25 ± 0.42 nmol/L (−1.07 to 0.57 nmol/L) lyso‐Gb3 +18 Da; −0.01 ± 0.21 nmol/L (−0.42 to 0.4 nmol/L) for lyso‐Gb3 +34 Da. The results showed a systematic positive bias of lyso‐Gb3, lyso‐Gb3 −28 and lyso‐GB3 −3 Da in capillary DBS most probably due to their presence in red blood cells.

Deming regression analysis was also conducted for HCT effects comparing plasma versus venous DBS and plasma versus venous Capitainer. The differences in the analogues concentrations were plotted as a function of HCT (34.3%–52.2%). Proportional bias was only observed for lyso‐Gb3 +18 Da (95% CI: 1.552–9.022 nM) for plasma versus venous DBS and lyso‐Gb3 +16 Da (95% CI: −2.853 to −0.357 nM) for plasma versus venous Capitainer. The analogues concentrations in the BµS did not change as a function of HCT. In all the comparisons, the coefficients of determination *R* ^2^ were <0.218 and the *p* values >0.001 for the slope hypothesis testing (that slope is zero). For this study, there was no correlation between the difference in concentration of analogues measured in plasma versus venous DBS and plasma versus venous Capitainer and HCT values of the study participants.

Results showed that Capitainer and DBS are feasible for screening, follow‐up and monitoring of patients with FB.

#### 4.2.3 Vitamins and Steroid Hormones

A relevant objective of the Binks et al. study was the evaluation of accuracy, bias and precision of 25‐hydroxyvitamin metabolites measurements in capillary DBS and venous DBS stored at 20°C for 2 months compared to plasma stored at −80°C for the same period of time [47]. 25‐Hydroxyvitamin D and its metabolites are used as biomarkers for vitamin D deficiency, bone health, autoimmune disease, cardiovascular disease and cancer [65, 66]. Plasma, venous DBS and capillary DBS samples were collected from 37 healthy volunteers and analysed using LC–MS/MS.

The mean 25OHD3 (major 25‐hydroxyvitamin metabolite) concentrations in the DBS matrices were similar, 68.7 nmol/L in venous DBS, 66.0 nmol/L in capillary DBS and lower compared to plasma 89.2 nmol/L. The linear correlation of 25OHD3 concentrations between venous DBS and capillary DBS was *R* ^2^ 0.92, venous DBS versus plasma was *R* ^2^ 0.93, and capillary DBS versus venous DBS *R* ^2^ 0.97. Ordinary least square regression showed that venous DBS and capillary DBS were very similar (*𝛃* = 1). However, there was a systematic difference between the DBS samples and plasma with (*𝛃* = 0.81). Bland–Altman analysis after bias correction (using the regression curve) showed that 95% of the values were within two SD of the mean difference. The precision was <15% CV. The data showed normal distribution per the Shapiro–Wilk test.

The results of this study indicated that a linking strategy is required between plasma and DBS data for accurate results on the levels of this metabolite.

In another study on vitamin D metabolites, Wang et al. developed a method for measuring 25OHD2 and 25OHD3 in DBS using LC–MS/MS [48]. As part of the validation study, comparison was performed for the 25OHD measurements in venous DBS, capillary DBS and plasma. In the first study, venous blood was randomly collected from individuals undergoing medical examinations and used to prepare the DBS and plasma samples. 25OHD3, a major vitamin D metabolite, was analysed in the two matrices, and a Passing–Bablok regression showed a slope of 0.982 (95% CI: 0.9167–1.0476) and intercept of 0.2324 (95% CI: −1.0571 to 1.0583). For linearity, a Cusum test showed no significant deviation (*p* = 0.37), and the Spearman rank correlation coefficient was 0.966 (95% CI: 0.944–0.980) with statistical significance (*p* < 0.0001). The Bland–Altman geometric mean ratio was 0.9818 (95% CI: 0.9528–1.0116). Ninety‐three per cent of samples passed the method comparison acceptance criteria of ±20% of the mean of both matrices. The acceptance criteria from the European Medicine Agency (EMA) guideline require at least 67%. 25OHD2 was compared in 11 samples, and 82% of the samples passed the criteria.

In order to further confirm the practicality of DBS in vitamins analysis, capillary DBS and plasma were collected from six individuals and compared. The bias ranged from −10.8% to −8.3% which is within the EMA ±20% criteria. The study showed that the two 25OHD metabolites can be accurately measured in DBS as compared to plasma.

In a follow‐up study, Wang et al. validated measurement of vitamin A using LC–MS/MS in DBS samples [49]. Vitamin A, also known as retinol, is a human metabolite that plays a role in vision, immune system, cellular growth and differentiation [67]. Excess retinol can also lead to toxicity with accompanying symptoms, including dizziness, headache and liver damage [68]. As part of the validation, 60 venous blood samples from randomly selected individuals undergoing medical examinations were used to prepare venous DBS and plasma samples for retinol measurement comparison. In a second experiment, plasma and capillary DBS from 12 individuals were used for a second method comparison experiment.

In the first study on venous DBS versus plasma, a Passing–Bablok regression showed a slope of 1.154 (95% CI: 1.0227–1.2970) and an intercept of −0.242 (95% CI: −0.4611 to −0.0459). A Cusum test for linearity showed no significant deviation with *p* = 1.00. The Spearman rank correlation coefficient *R* ^2^ was 0.888 (95% CI: 0.819–0.932) with statistical significance (*p* < 0.0001). The Bland–Altman showed a geometric mean ratio of 0.9804 (95% CI: 0.9495–1.0122). The results also showed that 90% of the paired (venous DBS vs. plasma) retinol samples had differences within the acceptance criteria (67% of samples within ±20% of the methods mean). For the second study on plasma versus capillary DBS retinol comparison, the results were even more comparable with only one sample outside the ±20% bias criteria.

In summary, the accurate and precise measurements of retinol in DBS show its potential for future routine use.

Steroids hormones, including testosterone (TES), epitestosterone, androstenedione (A4), progesterone (P), 17𝞪‐hydroxyprogesterone (17𝞪OHP), dehydroepiandrosterone (DHEA), dihydrotestosterone (DHT), corticosterone, cortisol, deoxycorticosterone and 11‐deoxycortisol, are routinely measured in athletes for antidoping evaluation [69]; thus, microsampling is a desirable approach in such cases. Salamin et al. compared measurement of endogenous steroids in serum, plasma and venous DBS [50]. Venipuncture blood collected from 100 elite athletes (51 female and 49 male) was used to prepare serum and plasma. DBS samples were spotted using the same venous blood samples and were all analysed using LC–MS/MS for steroids concentration comparison. The HCT effect in DBS measurements was normalized as follows: corrected concentration = (DBS concentration)/(1 − HCT).

In the TES's comparison between DBS and serum, the *R* ^2^ was 0.98, slope 0.68 (95% CI: 0.8–0.87) and intercept −0.03 (95% CI: −0.06 to 0.008), whereas in the comparison between DBS and plasma, the correlation was even higher with *R* ^2^ 0.99, slope 1.06 (95% CI: 1.05–1.1) and intercept 0.07 (95% CI: −0.12 to −0.03). In A4's comparison between DBS and the two conventional matrices, the *R* ^2^ was similar, 0.95. In A4's serum versus DBS comparison, the slope was 1.04 (95% CI: 0.95–1.15) and intercept 0.12 (95% CI: −0.09 to 0.31); in A4's DBS versus plasma comparison, the slope was 1.29 (95% CI: 1.2–1.42) and intercept −0.04 (95% CI: −0.15 to 0.21). Cortisol in venous DBS was underestimated in comparison with serum but had good agreement in plasma. 17𝞪OHP in venous DBS had good agreement with serum but was overestimated in comparison with plasma.

The highest steroids concentrations were observed in serum, followed by DBS, and the lowest in plasma. The results also indicate possible ion suppression due to the anticoagulants in the venous blood DBS samples. Proportional bias was observed for TES comparison between serum versus DBS and plasma versus DBS; for A4 in plasma versus DBS. No systematic bias was observed between venous DBS and the conventional matrices. The study highlighted the stability of metabolites in DBS samples as they had been in storage with desiccant for over 1 year before analysis. A limitation of the study could be the HCT effects when interpreting DBS concentrations using established reference ranges in conventional matrices. When a generic 45% HCT correction was applied between the matrices, there was no observed proportional difference for TES and A4 measurements.

The study showed that we can acquire accurate steroid concentrations from venous DBS samples compared to serum and plasma. However, studies featuring comparisons with capillary blood on DBS, the recommended collection, are required. Additionally, HCT corrections are required for interpretation of DBS concentrations using established reference ranges.

In a study of a different perspective, Ackermans et al. aimed to prove that HCT effects in DBS samples were inconsequential in the analysis of analytes that are mainly present in plasma [51]. Leftover venous blood samples (*n* = 30) were loaded on DBS, and the remaining was used for collection of plasma. 25OHD3 and TES were analysed in plasma and DBS samples using LC–MS/MS, and the concentrations of the two metabolites were compared.

Passing–Bablok regression was used to compare 25OHD3 and TES concentrations in HCT corrected and uncorrected DBS with plasma. The HCT correction transforms DBS concentrations to plasma concentrations so that established reference ranges can be used. The HCT correction was calculated as previously stated by Salamin et al. [50]. For 25OHD3, the non‐corrected HCT DBS concentrations versus plasma had an *R* ^2^ of 0.971, slope 0.74 (95% CI: 0.69–0.80) and intercept −2.20 (95% CI: −5.60 to 1.52); for the corrected‐HCT DBS versus plasma, the *R* ^2^ was 0.917, slope 1.24 (95% CI: 1.10–1.39) and intercept −1.84 (95% CI: −12.13 to 5.50). Regarding TES regression analysis for non‐corrected HCT DBS versus plasma, the *R* ^2^ was 0.994, slope 0.53 (95% CI: 0.51–0.57) and intercept −0.09 (95% CI: −0.12 to −0.04), whereas for corrected‐HCT venous DBS versus plasma, the *R* ^2^ was 0.953, slope 0.89 (95% CI: 0.83–1.00) and intercept −0.17 (95% CI).

The results showed that HCT correction for venous DBS samples was required in order to reduce bias; for both metabolites, the slope and intercepts were improved. Overall, the study highlights that the standardization of DBS results may be required for better interpretations of DBS results using plasma references or cut‐off values.

#### 4.2.4 Nucleotides

Nicotinamide adenine dinucleotide (NAD+) is an essential metabolite for energy production in cells and as such lower levels have been observed in the aging process of humans and rodents [70, 71, 72, 73]. Its precursors, including nicotinamide mononucleotide (NMN), have been shown to possess anti‐aging properties because they increase NAD+ levels. However, minimal studies have been conducted on the relationship between NAD+ and aging or aging‐related diseases due to its low stability in whole blood [74, 75].

In this study, Matsuyama et al. evaluated the measurements and stability of NAD+ and its precursor NMN in venous blood and DBS [52]. Venous blood was collected from healthy fasted individuals, and 5 µL was spotted on four types of DBS cards: Whatman FTA DMPK‐A, Whatman FTA DMPK‐B, Whatman FTA DMPK‐C and 903 protein saver cards. The stability of the analytes was first analysed in the remaining blood at day zero and in both samples matrices after 2 days, 1 week, 2 weeks and 1 month at 4°C storage. The mean ± SD concentrations of venous blood at day zero were 31.89 ± 6.43 and 1.83 ± 0.43 µM for NAD+ and NMN, respectively; the storage stability (mean ± SD) for NAD+ was 123.7% ± 23.8% (2 days), 134.9% ± 19.8% (1 week), 101.5% ± 17.8% (2 weeks) and 64.4% ± 5.9% (1 month). For NMN, the stability was 52.1% ± 20.4% (2 days), 27.8 ± SD not calculated due to levels below limit of quantitation (LOQ) (1 week), not calculated (2 weeks) and 65.7% ± 34.1% (1 month).

At 4°C, the NAD+ concentration increased in the first week, levelled after 2 weeks and decreased after 1 month. The temporary increase may be attributed to dephosphorylation of NADP which is also present in whole blood at refrigerated conditions [76, 77]. NMN degraded by ∼50% after 2 days and increased between 2 weeks and 1 month. This may be attributed to the degradation of NAD+ to NMN [52].

The stability of NAD+ was evaluated in the four DBS cards after storage at 4°C and room temperature. DMPK‐B had the highest stability (mean ± SD), 80.7% ± 11% (2 days), 87.8% ± 11.2% (2 weeks), 79.8% ± 13.9% (1 month) at 4°C and 82.0% ± 9.9% (2 days), 87.2% ± 21.4% (1 week) and 67.6% ± 4.6% (2 weeks) at room temperature. The stability in the other cards was in the following order: DMPK‐A > 903 protein saver card > DMPK‐C. The stabilities of NAD+ at room temperature after 2 days in the other cards were 71.6% (DMPK‐A), 55.9% (DMPK‐C) and 66.2% (903 protein saver cards). In comparison, the NAD+ was more stable in DMPK‐B after 1 week of storage at the same temperature conditions. Additionally, the stability in all DBS cards after 2 weeks was higher at 4°C than at room temperature.

For NMN, DMPK‐B also had the highest stability (mean ± SD), 95.4% ± 33.1% (2 days), 93.6% ± 38.1% (2 weeks) and 78.6% ± 40.2% (1 month) at 4°C. There was greater variability in the NMN concentrations compared to NAD+.

The study showed that NAD+ and NMN can be stored with high stability in DBS, particularly DMPK‐B paving a way for accurate analysis and aging‐related diseases research.

### 4.3 Comparison of BµS and Conventional Matrices in Markers of Toxicity and Abuse

In a study focusing on a toxicity biomarker, Menestrina Dewes et al. compared uracil measurements in TASSO + SST samples with those in plasma [32]. Uracil is a biomarker for 5‐fluorouracil (5‐FU) toxicity which is a chemotherapeutic agent used in cancer treatment. Accumulation of 5‐FU due to dihydropyrimidine dehydrogenase (DPD) enzyme deficiency can be fatal [78, 79, 80].

Uracil concentrations from TASSO + SST and plasma samples were compared using an LC–MS/MS assay. Forty‐one healthy volunteers (3 males and 38 females; average age 27.8) provided paired TASSO + SST serum and plasma samples. Four volunteers’ uracil concentrations were below the LOQ so only 37 of the 41 were compared.

Uracil measured concentration range in TASSO + SST samples was 5–21.9 ng/mL and in plasma 6.0–22.9 ng/mL. Passing–Bablok regression showed strong agreement in the two series of measurements with a rank correlation coefficient (*r*_s_) of 0.910 (*p* < 0.0001), slope 0.9731 (95% CI: 0.8726–1.1254) and intercept −0.008627 (95% CI: −1.9814 to 0.8517). Uracil concentrations were 2.3% higher in the plasma samples compared to TASSO + SST. The limits of agreement on the Bland–Altman plot ranged from −23.3% to 29.1%. Majority of the samples were within this range. The difference between the two techniques was within ±20% of the mean for 97% of the paired samples.

The study showed that TASSO + SST samples can be used for accurate measurements of uracil as compared to plasma. As uracil is not stable in blood, the TASSO + SST samples have to be collected at a health centre. Despite this, TASSO + SST offers convenient sample collection for adverse populations such as cancer patients.

There are also two studies [31, 34] investigating the use of BµS for the measurement of phosphatidylethanol (PEth). PEth is formed in blood in the presence of ethanol and is used as an alcohol abuse biomarker in forensics, toxicology and research [31, 81, 82]. The most abundant forms are PEth 16:0/18:1 (contain one palmitic and one oleic acid) and PEth 16:0/18:2 (contain one palmitic and one linoleic acid).

Jett et al. [34] focussed on the validation of TASSO‐M20 plugs in the measurement of PEth 16:0/18:1 compared to liquid venous blood. Comparison was conducted for 37 participants who had a partaken of alcohol during eight time intervals [34]. PEth analysis was performed using LC–MS/MS.

The first comparison for PEth concentrations was between TASSO‐M20 and liquid blood samples from 14 participants. A high agreement was found with a correlation *R* ^2^ of 0.988 and a slope of 0.951. The Bland–Altman bias and SD were 4.95% ± 11.3%; the 95% lower and upper limits were −17.2 to 27.1. The PEth levels in the TASSO‐M20 plugs were 5% higher in comparison to the liquid venous blood. Another correlation plot for a subset of the samples (seven participants) was prepared to evaluate the correlation of the sample matrices at the lower end of the concentration (0–200 ng/mL). PEth levels in the two matrices even at low concentration ranges were still highly correlated; *R* ^2^ was 0.944 and slope 0.816. The PEth concentrations between TASSO‐M20 and liquid blood were highly correlated, importantly at low concentration levels (<200 ng/mL). This shows TASSO‐M20's feasibility in the clinical care of individuals with alcohol use disorder where PEth measurements are routinely required.

In the other study, post‐sampling formation of PEth in blood due to presence of ethanol was compared between three BµS devices, DBS, Capitainer, Mitra and HDB, and liquid venous blood [31]. The BµS devices were spotted with 10 µL of 2 g/L ethanol‐spiked venous blood from 20 individuals and analysed using LC–MS/MS. It was observed that PEth was formed in all Capitainer samples (0.83–2.02 µmol/L), in almost half of the HDB samples (0.04–1.04 µmol/L) and in 8 out of the 20 Mitra samples in lower concentrations (0.02–0.12 µmol/L), whereas no formation of PEth was observed in DBS samples. On the other hand, PEth formation was observed in all blood samples (0.03–0.08 µmol/L). Additionally, post formation of PEth was not observed in Capitainer samples spiked with a sodium metavanadate (blocks phospholipase D enzyme required in PEth formation) inhibitor.

The results confirmed post‐sampling formation of PEth in liquid blood samples containing ethanol in contrast to DBS or other devices after the addition of inhibitor. Such a finding supports DBS's use for PEth analysis in order to avoid falsely elevated values which can have severe consequences on the subject being tested.

## 5 Untargeted Metabolomics Analysis

Untargeted metabolomics is the global measurement or profiling of all metabolites in cells, tissues or biological fluids in relation to genetic or external stimuli at a certain point in time [13, 83]. It can illustrate the complex phenotype and provide information on the metabolic state of the biological systems with regard to one's health, environment, lifestyle, diet and drug use, among other factors. It is therefore an impactful tool in biomarker discovery for disease diagnosis, prognosis and drug development, among others [13, 84]. This analysis is enabled by cutting‐edge MS techniques which can provide rich full information even with low amounts of volume. BµS analysis for metabolomics studies is still immature; thus, several studies exist investigating the advantages of this type of specimen over the conventional blood samples.

In the studies investigating global metabolic profiles of BµS devices against conventional matrices (venipuncture blood, plasma and serum) either capillary or venous blood was collected using DBS or Mitra devices (see Table 2). Unfortunately, the studies comparing capillary blood (the recommended collection) collected from fingertips on these devices are very limited (only two) [85, 86].

**TABLE 2:** Studies comparing global metabolic profiles in parallel by untargeted assays in blood microsamples (BµS) and liquid blood, plasma or serum.

Study type	Matrix/Microsampling device	Amount of sample	Analytical technique	Number of features detected	Comparative outcome	Study/Reference
Case–control study: Breast‐cancer metabolomic profiling	Venous DBS vs. venous Mitra vs. liquid venous blood	Venous DBS—8 µL × 5 spots, venous Mitra—10 µL × 4 tips, liquid venous blood—40 µL	GC–MS	Venous DBS ∼155, venous Mitra ∼153, **liquid venous blood ∼163** **Seven common metabolites were statistically different between the study groups in the three matrices**	In summary, Mitra and DBS performed equally well in terms of the number of detected metabolites, precision (Mitra's > DBS) and statistically significant metabolites found to discriminate between the patients group	[87]
Case–control study of HIV‐infected pregnant women	Venous DBS vs. plasma	DBS—2 × 6 mm^2^ punches, plasma—NA	LC–MS/MS	**Venous DBS and plasma: 627 common metabolites** **Only plasma: 260** Only venous DBS: 97 Total: 984	Although DBS and plasma showed some diverse information and had unique but also common features that could be potential biomarkers, the sample sizes in the study were small to draw impactful conclusions. Nonetheless, the selection of sampling techniques should be based on the feasibility of detecting biomarkers of interest and resources available	[88]
Investigating metabolic biomarkers of preterm births (PTB) among 100 pregnant women with HIV	Venous DBS vs. plasma	NA	LC–MS/MS	Random forest models utilizing DBS and plasma: untreated 95.5%, ZDV treated 95.7% and PI‐ART 80.7%	Although the study showed the feasibility of venous DBS in biomarker discovery of PTB in pregnant women living with HIV, it did not provide an extensive and accurate coverage of the metabolite profile in comparison with plasma	[89]
Plasma vs. venous DBS in application to diabetes mellitus	Venous DBS vs. plasma	Venous DBS—15 µL, plasma—100 µL	LC–MS/MS	POS mode: venous DBS 1295, **plasma 1869** **548 common metabolites** NEG Mode: Venous DBS 543, **plasma, 1191 plasma** **371 common metabolites**	The results showed that several metabolites in DBS had high correlation with plasma. Thus, for these specific ones, DBS, which offers a patient‐centric (fast and easy) approach, could be used	[90]
Case study: The potential of multiomics microsampling in lifestyle associated health	Venous Mitra vs. plasma	Venous Mitra—10 µL, plasma—NA	LC–MS/MS	∼560 metabolites, ∼155 lipids, ∼54 cytokines/hormones in both matrices	The results showed that most of the metabolites found in plasma were also present in the Mitra samples and at high correlations	[91]
Integrated metabolomics of different DBS matrices and plasma	Capillary DBS vs. venous DBS vs. plasma DBS vs. plasma	Venous DBS—3 mm, plasma—NA	LC–MS/MS and GC–MS	>300 in all matrices	The authors concluded that the molecular species identified in DBS by the applied analytical platforms have the potential to deliver valuable insight for the application of personal metabolome monitoring	[86]
Comparison of global metabolic profiles of healthy fasted individuals using three microsampling devices and plasma	Venous DBS vs. venous Mitra vs. venous Capitainer vs. plasma	20 µL	LC–MS/MS	**Venous Capitainer—7403** Venous Mitra—6945 Venous DBS—6846 Plasma—4455 **The largest number of common features with plasma was with Mitra—105**	The results showed that BµS devices, Capitainer and Mitra, were able to capture equal or even greater information (for some metabolites most probably in red blood cells) on global metabolic profiles compared to plasma. Despite the preliminary results, a large‐scale study with in‐depth investigation of each BµS device using capillary blood is required to decipher their differences with plasma	[92]
Comparative study: capillary Mitra vs. venous Mitra vs. plasma Mitra	Venous Mitra, Capillary Mitra and plasma Mitra	NA	LC–MS/MS	Capillary Mitra ∼132 **Venous Mitra ∼155** Plasma Mitra ∼37	In general capillary blood provided superior coverage of the polar metabolome which included intracellular metabolites from red blood cells and in addition was able to capture sex‐differentiating metabolites	[85]
Effects of different storage conditions on metabolic profiles of venous blood collected on Mitra and DBS against plasma samples	Venous DBS, venous Mitra and plasma	Venous DBS 5 mm punch (∼10 µL), Mitra 10 µL and plasma 10 µL	LC–MS/MS	Features detected at different storage conditions and time: 1 day at −80°C: the lowest number was in plasma 81%–84% 168 days at −80°C: 77% venous DBS, 69% venous Mitra and 65% in plasma. 168 days −20°C: 80% venous DBS, 74% Mitra, 47% in plasma	The results suggest that BµSs are viable substitutes for plasma when collection of the latter is not feasible, for example in remote geographical regions and out of clinic settings	[93]

Abbreviations: DBS, dried blood spot; GC–MS, gas chromatography–mass spectrometry; LC–MS, liquid chromatography–mass spectrometry; PI‐ART, protease‐inhibitor based antiretroviral therapy; ZDV, zidovudine monotherapy.

### 5.1 Comparison of BµS and Conventional Matrices in Case–Control Biomarker Discovery Studies

Cala et al. compared the metabolic profiles of 10 controls (CP) and 10 breast cancer patients (BCP) using venous blood sampled on DBS and Mitra, against liquid blood [87]. The performance of the three matrices was evaluated by comparing, as criteria, the number of detected metabolites, the precision of signal intensities and the level of information to classify the groups by univariate (UVA) and multivariate (MVA) analysis. Analysis was performed using GC–MS/MS.

As a first finding, very similar numbers of identified metabolites were noted in the three matrices: DBS (155), Mitra (153) and liquid blood (163). In comparison to blood, 94% of the metabolites were identified in DBS and 93% in the Mitra. After applying the 80% rule and 30% CV cut‐off, the numbers of metabolites were DBS (73), Mitra (81) and liquid blood (84). Mitra showed higher precision than DBS which could be attributed to its ability to collect a defined volume of blood; QC samples clustered in all individual matrix PCA models with liquid blood showing the tightest cluster, therefore, indicating the highest precision. PCA showed discrimination of CP from BCP in all analysed matrices. Supervised MVA analysis was also performed to identify biomarkers discriminating between the two groups in each matrix. All three matrices showed clear separation in the OPLS‐DA models which were cross‐validated to assess predictability. The predictive abilities of the OPLS‐DA models were 97% for liquid blood, 96% for DBS and 96% for Mitra.

The results showed that DBS and Mitra can yield almost equal results in GC–MS metabolic fingerprinting when compared to liquid blood. Further analysis determined that 11, 12 and 9 metabolites were significantly different in the liquid blood, DBS and Mitra OPLS‐DA models, respectively. Fourteen total metabolites were significantly differentiated in the study groups taking into account all the three matrices. Of the 14 significant metabolites, 7 (fatty acids, amino acids and derivatives, and organic acids) were detected in all three matrices. Tryptophan was uniquely detected in Mitra; linoleic acid, arachidonic acid and cholesterol in liquid blood and DBS.

In summary, Mitra and DBS performed equally well in terms of number of detected metabolites, precision (Mitra's was better than DBS) and statistical significance of metabolites found to discriminate between the two groups.

Tobin et al. study compared the use of venous DBS and plasma metabolomics for predictive modelling and biomarker discovery in pregnant women with HIV [88]. In this study, 79 pregnant women living with HIV were either untreated or undergoing two different treatments. Analysis was performed using four LC–MS/MS assays (Reverse Phase and HILIC‐MS in POS and NEG mode). A total of 627 (63.7%) metabolites were detected in both DBS and plasma, 260 (26.4%) in plasma and 97 (9.9%) in DBS. In total, 984 metabolites were detected. A permutational multivariate analysis of variance (PERMANOVA) of the paired DBS and plasma samples accredited 69% (*p* < 0.001) of the variation to the individual. The mean intraclass correlation coefficient (ICC) was 0.52. The distances between DBS and plasma samples for the same women were significantly smaller compared to distances between different women in the study (Wilcoxon *p* < 0.001).

The ICC of the metabolites between the two matrices was analysed. Some metabolites (212) had good ICC (l 0.75), whereas 121 metabolites had excellent ICC (s 0.9). Metabolic profiles of the pregnant women in the three different groups (untreated, zidovudine monotherapy [ZDV] and protease‐inhibitor‐based antiretroviral therapy [PI‐ART]) were analysed using random forest classification. The DBS models’ accuracies were 90.9%, 95.7% and 87.1% for the untreated, ZDV and PI‐ART groups, respectively. The plasma model accuracies were lower, 86.4%, 91.3% and 77.4% in the same groups. There was a major difference in the features identified in the DBS and plasma models. Only 20/147 features identified in either model were identified in both DBS and plasma models for the same treatment. These compounds included sulphated steroid compounds and methionine metabolism products (methionine sulphone and *N*‐methylmethionine).

Although DBS and plasma showed some diverse information and had unique but also common features that could be potential biomarkers, the sample sizes in the study were small to draw impactful conclusions. Nonetheless, the selection of sampling techniques should be based on the feasibility of detecting biomarkers of interest and resources available.

In a follow‐up study, Tobin et al. investigated the metabolic biomarkers of preterm births (PTB) among 100 pregnant women with HIV [89]. Again, the same three groups were compared, untreated: treated with ZDV or treated with protease PI‐ART. For controls, women who had PTB were matched by gestational age at sample collection time. Venous blood was collected from preterm women between 25 and 35 weeks of gestation before or during treatment. The blood was spotted on DBS cards and the remaining separated for plasma collection. Analysis was conducted similar to the previous study.

Random forest models utilizing both plasma and DBS were evaluated for each group. The accuracies of the models were 95.5%, 95.7% and 80.7% for the untreated, ZDV and PI‐ART, respectively. In the untreated group, urate and *N*‐acetyl‐1‐methylhistidine in plasma were determined as biomarkers of PTB; dopamine 3‐*O*‐sulphate, methionine sulphone and allantoin were identified as significant in DBS. In the ZDV group, methionine sulphone and hippurate were significantly different in both plasma and DBS, whereas 17𝞪‐hydroxypregnenolone glucuronide was in the former. In the PI‐ART group, 17 metabolites, including steroids such as cortisone, were significantly different. In the DBS samples, only two metabolites were significant, 7‐methylguanine and *N*2,*N*2‐dimethylguanosine.

Although the study showed the feasibility of venous DBS in biomarker discovery of PTB in pregnant women living with HIV, it did not provide an extensive and accurate coverage of the metabolite profile in comparison with plasma.

In a study by Chiu et al., the global metabolic profiles of venous DBS and plasma samples of diabetes mellitus cases (*n* = 32) and controls (*n* = 32) were analysed by LC–MS/MS [90]. Different volumes were used for analysis: 15 µL of venous blood spotted on DBS cards and 100 µL of plasma. For that reason, an optimized extraction protocol was used for each matrix based on the author's literature search.

It was shown that despite the difference in volumes, a total number of 1295 features were identified in DBS and 1869 in plasma in positive mode; the respective numbers in negative mode were 543 and 1191. The numbers of common features between the matrices were 748 and 371 in positive and negative modes, respectively. Regression analysis performed between the features intensities identified in both matrices showed that of the annotated features, 35 had high correlation (*R* ^2^ > 0.7), 14 had moderate correlation (*R* ^2^ between 0.4 and 0.7), and 25 low correlation (*R* ^2^ < 0.4). Metabolites that showed high correlation between the matrices included cholic acid‐derived metabolites, xanthines, hydroxybutyric acids, hippuric acids, some carnitines and multiple amino acids and their derivatives. Unsupervised PCA analysis showed clear discrimination between DBS and plasma features on PC1. Metabolites such as 5′‐methylthioadenosine and creatine were higher in DBS than in plasma indicating that they may be mainly found in red blood cells.

Supervised PLS DA models of each matrix comparing the cases and controls did not show clear separation. However, 2‐hydroxybutyric acid and hexoses were significantly different in the cases and controls with a false discovery rate (FDR)‐adjusted *p* < 0.05 in both plasma and DBS. 2‐Hydroxybutyric acid had a strong correlation (*R* ^2^ 0.96) and hexoses a moderate one (*R* ^2^ 0.55) between plasma and DBS.

The results showed that several metabolites in DBS had high correlation with plasma. Thus, for these specific ones, DBS which offers a patient‐centric (fast and easy) approach could be used.

### 5.2 Comparison of BµS and Conventional Matrices in Lifestyle Health Studies

In a large multi‐omics study with a main aim to assess response to dietary intervention and to reveal intraday molecular fluctuations [91], metabolomics profiles of 34 individuals were acquired using venous blood collected on Mitra and plasma. The comparison study aimed to demonstrate microsampling potentials in dynamic health profiling and biomarker discovery. Analysis was performed using four assays, RP and HILIC‐MS (both in positive and negative modes). The features detected were as follows: 7487 in RP POS, 4662 in RP NEG, 6362 in HILIC POS and 4374 in HILIC NEG. The metabolites identified using Mitra had a high correlation with those in plasma, and the Spearman correlation (*ρ*) was 0.8–0.9. The *ρ* for 642 metabolites was 0.81 (*p* < 0.001) and 0.94 (*p* < 0.001) for 616 lipids. Most of the amino acids, carbohydrates, free fatty acids, triacylglycerols, diglycerides and phosphatidylcholines had the highest correlations between Mitra and plasma.

The results showed that most of the metabolites found in plasma were also present in the Mitra samples and at high correlations.

### 5.3 Comparison of Different Types of BµS With Plasma in Studies With Healthy Volunteers

In one study, the main focus was on the stability of metabolites in dried samples; however, an additional focus was applied to the metabolic profiles comparisons of capillary and venous blood dried samples with plasma [85]. Four different types of samples were investigated: Capillary DBS (from finger), venous DBS, plasma DBS and plasma were collected from 16 healthy individuals (eight male and eight female [mean age 32 ± 6 years]) after an overnight fast. The analysis was performed using HILIC‐MS/MS and GC–MS. With the applied platform, a list of 428 endogenous analytes could be targeted in human biofluids. From these, about 300 metabolites were detected in the blood samples with plasma showing the highest coverage. In comparison to plasma, capillary DBS was missing 36, venous DBS 22 and plasma DBS 11 metabolites. However, approximately 15% of the metabolites detected in DBS were not detected in liquid plasma. They included adenosine 5′‐triphosphate, 5‐methyltetrahydrofolic acid and iso‐citric acid, among other phosphates.

The authors concluded that the molecular species identified in DBS by the applied analytical platforms have the potential to deliver valuable insight for the application of personal metabolome monitoring.

In a very recent study from our group, the global metabolic profiles of venous blood from 10 individuals on three different devices, Capitainer, Mitra and DBS, versus plasma samples were compared [92]. Venous blood was collected from 10 (five men and five women) healthy, overnight‐fasted individuals and 10 µL were loaded on each of the devices, whereas the rest was used for plasma collection. The samples were analysed using an LC–MS/MS in RP positive mode.

The three BµS devices provided richer profiles with an average of 7000 features detected compared to the 4455 features of plasma. Common features between each of the BµS devices and plasma were as follows: Capitainer and plasma 11, Mitra and plasma 105, and DBS and plasma 12. Of the 46 annotated metabolites, the largest number with highest intensity were observed in Mitra (31 metabolites) and second in plasma (14 metabolites). Analysis of variance (ANOVA) of the four matrices showed that eight of the metabolites were statistically different (*p* < 0.05) in the analysis, including food biomarkers trigonelline, theobromine, theophylline and caffeine; amino acids and derivatives proline, tyrosine, hippuric acid; a lipid, glycocholic acid. Another major observation was lack of detection of key metabolites in plasma, including ergothioneine, glutathione reduced, glutathione oxidized, s‐4‐(2‐*oxo*‐butyl)glutathione and cysteinylglycine compared to the BµS.

A PCA plot showed a clear discrimination of the plasma global metabolic profiles from the three BµS devices on PC1 (0.513) and PC2 (0.148). MVA analysis was used to evaluate the level of information acquired by the different matrices regarding the genders of the volunteers. Separation of males from females global metabolic profiles was only observed in plasma samples, though at an insignificant level.

The results showed that BµS devices, mainly Capitainer and Mitra, were able to capture equal or even greater information (for some metabolites, most probably in red blood cells) on global metabolic profiles compared to plasma. Despite the preliminary results, a large‐scale study with in‐depth investigation of each BµS device using capillary blood is required in order to decipher their differences with plasma.

Lastly, there was a study that did not involve any analysis of liquid blood or plasma. However, it was included in this review as it provides important information on the differences observed in terms of the metabolites found in venous blood, capillary blood and plasma collected on Mitra [86].

In this study, three types of blood were collected on Mitra: finger capillary blood, venous blood and plasma. The samples were collected from 22 healthy volunteers (11 male and 11 female whose ages matched) in duplicate (*n* = 2 per sample type). Analysis was performed by HILIC‐MS in both positive and negative mode covering hydrophilic metabolites.

MVA analysis (PCA) of the polar metabolome showed clustering based on sample type in both modes. The coverage of the metabolome differed. Venous Mitra had the highest number of features detected followed by capillary blood Mitra and lastly, by plasma Mitra in both modes. According to the authors, this was expected as the analytical method covers intracellular metabolites present in red blood cells [94, 95, 96].

In positive mode, a total of 206 features were detected in all three sample types, whereas some metabolites were unique: 76 in venous Mitra, 43 in capillary Mitra and 13 in plasma Mitra samples. The largest overlap of features (292) was seen in venous and capillary blood. Similar findings were also acquired by negative mode data.

**FIGURE 3:**
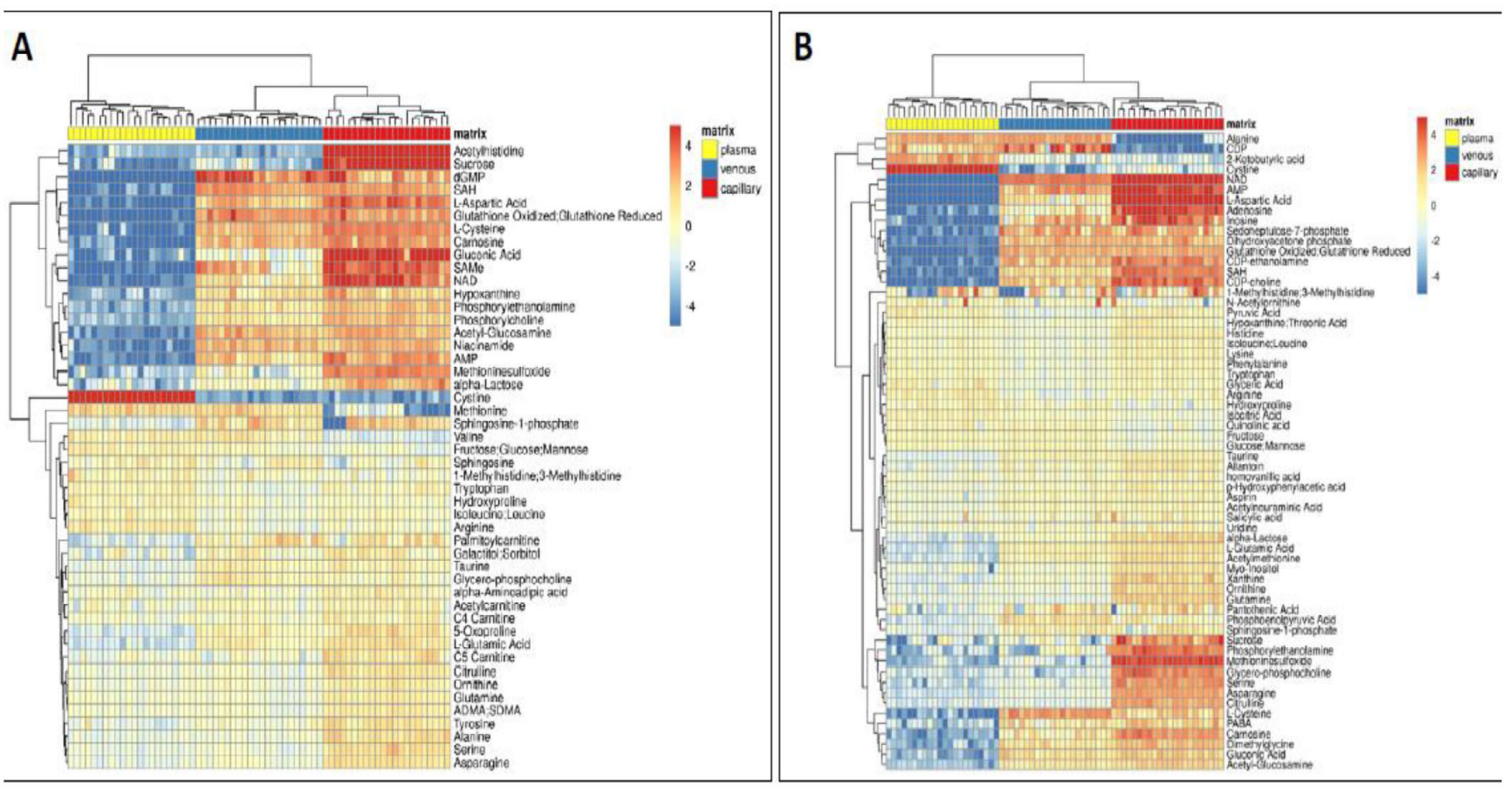
Matrix plots showing the quantitative comparison of annotated metabolites (in positive mode: A and negative mode: B) in three sample types: plasma, blood and capillary blood, all sampled on Mitra. NAD, nicotinamide adenine dinucleotide *Source*: Volani et al. [86].

Some intracellular metabolites were only identified in capillary and venous dried blood, whereas those that were present in all sample types showed variation. In Figure 3, differences in the abundance of metabolites identified in the three different types of samples (plasma Mitra, venous blood Mitra and capillary blood Mitra) can be seen [86]. As examples, intracellular metabolites such as *S*‐adenosylhomocysteine, glutathione and phosphorylcholine were largely detected in venous and capillary blood. Most of the amino acids such as arginine, valine and cystine had higher intensities in plasma possibly due to their fast metabolism or transportation through the cell walls [97]. However, citrulline had higher intensity in capillary Mitra. On the other hand, glucose had higher intensity in plasma possibly due to glycolysis in the cells.

On the basis of the obtained features, statistical analysis was performed to explore whether the data from the three sample types could equally classify the two genders. The *ρ* of log2‐fold change values was 0.66 between venous and capillary blood and 0.53 between capillary and plasma in positive mode; it was 0.61 and 0.42 in negative mode, respectively. Few features were significantly different in the samples after hypothesis testing adjustment. For example, gluconic acid (intracellular) which is a product of glucose oxidation had higher intensity in males in venous and capillary blood compared to plasma due to low concentration in plasma [98]. All significant features followed the same trend in the three matrices (higher in capillary and venous, and lower in plasma) except for a few such as aspartic acid which was detected at a very low level in plasma.

In general, capillary blood provided superior coverage of the polar metabolome which included intracellular metabolites from red blood cells, and in addition was able to capture sex‐differentiating metabolites.

### 5.4 Evaluation of the Effects of Storage Time and Temperature on Metabolic Profiles of Different BµS and Plasma

A study was designed specifically to investigate the effects of different storage conditions on metabolic profiles of venous blood collected on Mitra and DBS against plasma samples [93]. Venous blood was collected from one volunteer and was used for the preparation of the three types of samples. Analysis was conducted using RP and HILIC LC–MS/MS assays in positive and negative modes, whereas storage conditions examined were 4°C, −20°C and −80°C for up to 6 months.

After 1 day of storage at −80°C, the lowest numbers of features were identified in plasma (84% in positive and 81% in negative mode) in comparison with venous Mitra and DBS. 4‐Acetamidobutanoic acid and guanidino‐succinic acid were not detected in DBS, whereas 4‐hydroxybenzoic acid, ophthalmic acid, histamine and methylparaben were not detected in plasma.

Two‐way ANOVA models with time and temperature interaction plus RSD were used to classify stability of annotated metabolites by storage conditions. Only 119 metabolites detected in more than half of all samples were considered. From the metabolites that were not significantly affected by time and temperature, 38% were fully stable in Mitra, 39% in DBS and only 17% in plasma. It was also found that in plasma more metabolites, 41%, showed a time‐temperature interaction compared to 21% in DBS, and 17% in Mitra showed the effect of storage time varied by temperature.

After 168 days and at −80°C, the highest percentage of stable metabolites was measured in DBS (77%), Mitra (69%) and then in plasma (65%); at −20°C, 80% metabolites were stable in DBS, 74% in Mitra and 47% in plasma; at 4°C, the highest number of stable metabolites was in Mitra (81%). It was also observed that lipid metabolites levels increased in warmer storage conditions in plasma and DBS samples, whereas amino acids decreased or increased evenly in all the matrices. At 4°C, some metabolites and lipids (glycerophosphocholine) displayed larger log2‐fold changes over time in plasma compared to the other two matrices, whereas at −20°C, some lipids (fatty acids and glycerophosphocholines) displayed small increases in log2‐fold changes over time in plasma but not in Mitra and DBS.

The results suggest that BµSs are viable substitutes for plasma when collection of the latter is not feasible, for example, in remote geographical regions and out of clinic settings.

## 6 Discussion

BµS techniques that enable minimal invasive self‐sampling and support remote and longitudinal clinical research ultimately can greatly aid in biomarker discovery, clinical screening and diagnosis thus improving patient health. In order to fully implement such an approach in the clinical and research fields, accurate and precise validation and parallel studies against conventional matrices are required.

This review examines recent studies conducted in pursuit of this aim, indicating increased interest in microsampling for biomarkers analysis. For the review, 11 different BµS devices were used, seven of which collect and store dried blood (DBS, Capitainer, Mitra, hemaPEN, TAP II, TASSO‐M20 and HDB) and four which collect liquid capillary blood or serum (TASSO+, TASSO + SST, OSG and BDµM).

**FIGURE 4:**
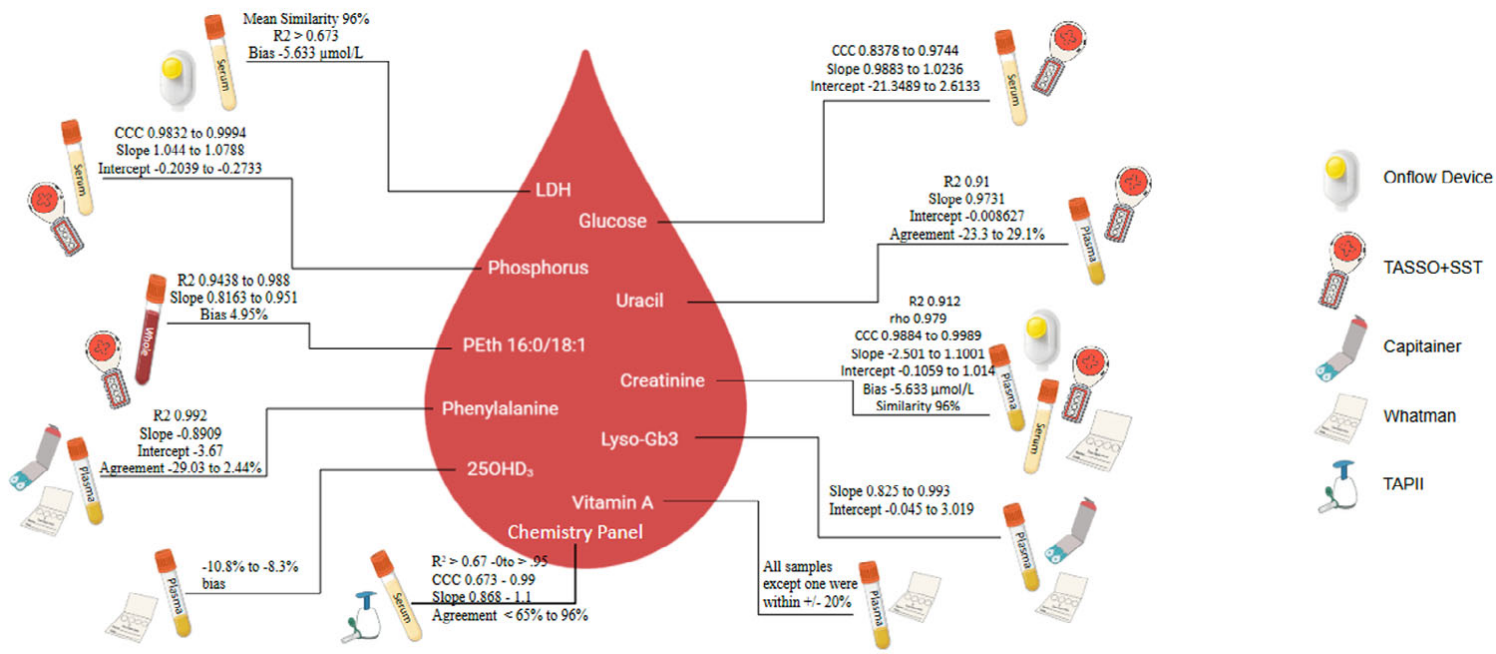
Diagram showing the comparison results (*R* ^2^, CCC, slope, intercept, % bias and agreement) between different capillary BµS and conventional matrices. CCC, concordance correlation coefficient; LDH, lactulose dehydrogenase; PEth, phosphatidylethanol; SST, serum separator tube; TAP II, Touch Activated Phlebotomy.

Although dried BµS devices are designed for capillary blood collection, most studies have so far focused on venous blood. Only a few studies comparing capillary BµS were solely analysed in parallel with conventional matrices or in combination with venous BµS [46, 47, 48, 49, 85, 86]. In Figure 4, the metabolites that were measured in capillary blood collected from either the upper arm or the fingertip using various BµS devices (OSG, TASSO + SST, Capitainer, DBS and TAP II) in parallel to conventional blood samples are graphically depicted. In the cases where the same samples in dried and liquid form are compared, differences revealed would most likely be primarily related to analyte stability, inefficient extraction from the materials and constraints associated with smaller sample volumes. However, in cases where venous dried blood is compared with plasma or serum, additional differences may arise as whole blood contains metabolically active cells that store, consume or alter metabolites, whereas plasma only reflects the cell‐free, soluble fraction. Therefore, expanding comparisons between plasma or serum and true capillary samples presents a valuable opportunity to support BµS future clinical integration. The majority of the studies indicated that venous BµS can acquire equal information compared to conventional matrices, with authors acknowledging that comparisons with capillary blood are required to further confidently showcase the viability of BµS in the clinical and research fields [31, 34, 41, 42, 45, 48, 49, 50, 51, 52].

In most of the studies, agreement of measurements was assessed for a small number of samples (ranging from 12 to 51). There were also cases where more than 100 samples were measured [41, 43]. For analysis between capillary BµS and conventional matrices, agreement was shown in most of the cases by regression analysis, Bland–Altman plot assessing % bias or CCC. Fitness in the regression models of the applications presented here ranges from *R* ^2^ of 0.944 (for PEth) to 0.992 (for phenylalanine). Biases range from −13.3% (for phenylalanine) to 14.7% (for LDH), whereas CCCs were from 0.673 (for LDH) to 0.99 (for creatinine). For the comparison results (*R* ^2^, CCC, slope, intercept, % bias and agreement) between different capillary BµS and conventional matrices, see Figure 4 [33, 34, 35, 44, 47].

A major bottleneck in the implementation of BµS in routine clinical analysis is the establishment of reference ranges for these specific matrices as traditional reference ranges have long been established for conventional matrices such as plasma, serum and liquid blood. In order to interpret metabolite concentrations from BµS, additional steps are required. As an example, creatinine, an important kidney biomarker, is measured only in plasma and serum. In some studies presented here, regression correction and conversion factors (determined from regression analysis with a conventional matrix) were used to correct for systematic bias of measurement in BµS [41, 42]. Similar cases were shown for measurement of 25OHD3 and TES [51] in BµS. Until reference ranges are established for biomarker analysis in BµS, such corrections are required to ensure accurate interpretation in capillary blood.

Another important takeaway from the studies is the sample size and health status of the participants involved. Although good correlations and within range % bias (per authors selected laboratory guidelines) were observed between BµS and conventional matrices, further studies involving larger and more diverse participant groups with varying health statuses and broader analytical reference ranges are needed to increase confidence in the results [2, 33, 35].

Finally, regarding analytical technologies, MS methods were mainly used, for example, for analysis of amino acids and their derivatives, carnitines, lipids species, vitamins, steroids, nucleotides and alcohol markers of abuse in metabolite biomarker analysis; this was also the case for global metabolic profiling analysis. Immunoassays were featured in a few of the articles for chemistry panel metabolites, including LDH, glucose, phosphorus, BIL, BUN, cholesterol, carbon dioxide, creatinine, glucose, HDL and TRIG. It should be stressed that newly developed methods for the analysis of BµS require thorough validation. In the studies presented here, method validation studies were conducted for the analysis of uracil, creatinine, globotriaosylsphingosine (lyso‐Gb3) and its analogues, 25OHD2 and its metabolites, and vitamin A using various BµS devices in accordance with established laboratory guidelines [32, 41, 43, 46, 47, 48, 49]. However, in the rest of the applications, for example, for PEth, amino acids and derivatives, carnitines and steroids, no validation steps were reported [34, 42, 44, 45, 50]. Nonetheless, validation experiments following existing guidelines though originally designed for traditional matrices such as whole blood, plasma or serum are essential prior to the implementation of BµS analysis in routine clinical practice. These must also account for additional parameters, such as sample volume, HCT effects and stability, which are often overlooked or inadequately assessed [99].

## 7 Conclusions

Interest in the application of metabolomics toward clinical diagnostics and population health monitoring has grown. Microsampling represents a major technique in the realization of these goals. In order for microsampling to be adopted in clinical routine and biomarker research, enhanced comparison and validation studies in comparison with conventional matrices are required. Future research is also very likely to focus on its integration in multi‐omics studies (genomics, proteomics, metabolomics, lipidomics) that could enable more comprehensive profiling from minimal sample volumes. Combination with digital health technologies may even enhance real‐time monitoring and personalized medicine applications.

Current research has shown great promise in this direction, as BµS analysis allows precise and accurate results in metabolite biomarker analysis, similar to the conventional matrices. Depending on the analyte, however, inconsistencies observed require correction and conversion of metabolite concentrations in BµS before any interpretation using current established ranges. With regard to untargeted metabolomics, the reviewed literature has shown that BµS can achieve equal or even greater information on global metabolic profiles; however, the choice of device should be determined by biomarkers of interest because metabolite‐dependent trends are evident.

Although the reviewed data suggest that BµS can be implemented in routine clinical analysis and biomarker research, more studies incorporating capillary BµS (the recommended blood type for BµS) as opposed to venous BµS are required. Additionally, larger study groups encompassing healthy and diseased participants covering wider metabolite concentration ranges should be used for higher validation confidence.

## Author Contributions

**Helen Gika**: conceptualization, methodology, writing – review and editing, supervision. **Marlene Thaitumu**: methodology, investigation, writing – original draft preparation, writing – review and editing. **Vasiliki Gkanali**: writing – original draft preparation, writing – review and editing. **Georgios Theodoridis**: supervision. All authors have read and agreed to the published version of the manuscript.

## Consent

The authors have nothing to report.

## Conflicts of Interest

The authors declare no conflicts of interest.

## Supporting information




**Supporting File 1**: ansa70044‐sup‐0001‐SuppMat.docx

## Data Availability

The authors have nothing to report.

## References

[1] M. U. Thangavelu , B. Wouters , A. Kindt , I. K. M. Reiss , and T. Hankemeier , “Blood Microsampling Technologies: Innovations and Applications in 2022,” Analytical Science Advances 4, no. 5–6 (2023): 154–180, 10.1002/ansa.202300011. 38716066 PMC10989553

[2] B. B. Collier , W. C. Brandon , M. R. Chappell , P. M. Kovach , and R. P. Grant , “Maximizing Microsampling: Measurement of Comprehensive Metabolic and Lipid Panels Using a Novel Capillary Blood Collection Device,” Journal of Applied Laboratory Medicine 8, no. 6 (2023): 1115–1126, 10.1093/jalm/jfad066. 37748757

[3] N. Grüner , O. Stambouli , and R. S. Ross , “Dried Blood Spots–Preparing and Processing for Use in Immunoassays and in Molecular Techniques,” Journal of Visualized Experiments: JoVE 2015, no. 97 (2015): 52619, 10.3791/52619. PMC4397000 25867233

[4] R. Guthrie and A. Susi , “A Simple Phenylalanine Method for Detecting Phenylketonuria in Large Populations of Newborn Infants,” Pediatrics 32 (1963): 338–343. 14063511

[5] I. R. Müller , G. Linden , M. F. Charão , M. V. Antunes , and R. Linden , “Dried Blood Spot Sampling for Therapeutic Drug Monitoring: Challenges and Opportunities,” Expert Review of Clinical Pharmacology 16, no. 8 (2023): 691–701, 10.1080/17512433.2023.2224562. 37300458

[6] M. Perilli , F. Toselli , L. Franceschetto , et al., “Phosphatidylethanol (PEth) in Blood as a Marker of Unhealthy Alcohol Use: A Systematic Review With Novel Molecular Insights,” International Journal of Molecular Sciences 24, no. 15 (2023): 12175, 10.3390/ijms241512175. 37569551 PMC10418704

[7] Y. Yuan , Y. Xu , and J. Lu , “Dried Blood Spots in Doping Analysis,” Bioanalysis 13, no. 7 (2021): 587–604, 10.4155/bio-2021-0019. 33728984

[8] S. Gaugler , J. Rykl , I. Wegner , T. von Däniken , R. Fingerhut , and G. Schlotterbeck , “Extended and Fully Automated Newborn Screening Method for Mass Spectrometry Detection,” International Journal of Neonatal Screening 4, no. 1 (2017): 2, 10.3390/ijns4010002. 33072928 PMC7548895

[9] Z. H. Gong , G. L. Tian , Q. W. Huang , Y. M. Wang , and H. P. Xu , “Reduced Glutathione and Glutathione Disulfide in the Blood of Glucose‐6‐Phosphate Dehydrogenase‐Deficient Newborns,” BMC Pediatrics [Electronic Resource] 17, no. 1 (2017): 172, 10.1186/s12887-017-0920-y. 28728551 PMC5520230

[10] F. A. Zubaidi , Y. M. Choo , G. H. Tan , H. A. Hamid , and Y. K. Choy , “A Novel Liquid Chromatography Tandem Mass Spectrometry Technique using Multi‐Period‐Multi‐Experiment of MRM‐EPI‐MRM3 With Library Matching for Simultaneous Determination of Amphetamine Type Stimulants Related Drugs in Whole Blood, Urine and Dried Blood Stain (DBS)‐Application to Forensic Toxicology Cases in Malaysia,” Journal of Analytical Toxicology 43, no. 7 (2019): 528–535, 10.1093/jat/bkz017. 31141150

[11] I. G. Herrera‐Pérez , A. S. Rodríguez‐Báez , A. Ortiz‐Álvarez , et al., “Standardization and Validation of a Novel UPLC‐MS/MS Method to Quantify First Line Anti‐Tuberculosis Drugs in Plasma and Dried Blood Spots,” Journal of Chromatography. B, Analytical Technologies in the Biomedical and Life Sciences 1228 (2023): 123801, 10.1016/j.jchromb.2023.123801. 37453389

[12] M. Okano , A. Miyamoto , M. Ota , S. Kageyama , and M. Sato , “Doping Control Analysis of Trimetazidine in Dried Blood Spot,” Drug Testing and Analysis 16, no. 8 (2024): 766–776, 10.1002/dta.3414. 36417202

[13] H. Gika , C. Virgiliou , G. Theodoridis , R. S. Plumb , and I. D. Wilson , “Untargeted LC/MS‐Based Metabolic Phenotyping (Metabonomics/Metabolomics): The State of the Art,” Journal of Chromatography. B, Analytical Technologies in the Biomedical and Life Sciences 1117 (2019): 136–147, 10.1016/j.jchromb.2019.04.009. 31009899

[14] M. G. M. Kok and M. Fillet , “Volumetric Absorptive Microsampling: Current Advances and Applications,” Journal of Pharmaceutical and Biomedical Analysis 147 (2018): 288–296, 10.1016/j.jpba.2017.07.029. 28803682

[15] C. Stove and N. Spooner , “DBS and Beyond,” Bioanalysis 7, no. 16 (2015): 1961–1962, 10.4155/bio.15.139. 26327175

[16] D. He , Q. Yan , K. Uppal , et al., “Metabolite Stability in Archived Neonatal Dried Blood Spots Used for Epidemiologic Research,” American Journal of Epidemiology 192, no. 10 (2023): 1720–1730, 10.1093/aje/kwad122. 37218607 PMC11004922

[17] A. M. Dijkstra , P. de Blaauw , W. J. van Rijt , et al., “Important Lessons on Long‐Term Stability of Amino Acids in Stored Dried Blood Spots,” International Journal of Neonatal Screening 9, no. 3 (2023): 34, 10.3390/ijns9030034. 37489487 PMC10366855

[18] G. Nys , M. G. M. Kok , A. C. Servais , and M. Marianne Fillet , “Beyond Dried Blood Spot: Current Microsampling Techniques in the Context of Biomedical Applications,” TrAC 97 (2017): 326–332, 10.1016/j.trac.2017.10.002.

[19] B. U. W. Lei and T. W. Prow , “A Review of Microsampling Techniques and Their Social Impact,” Biomedical Microdevices 21, no. 4 (2019): 81, 10.1007/s10544-019-0412-y. 31418068 PMC6695349

[20] P. Couacault , D. Avella , S. Londoño‐Osorio , et al., “Targeted and Untargeted Metabolomics and Lipidomics in Dried Blood Microsampling: Recent Applications and Perspectives,” Analytical Science Advances 5, no. 5–6 (2024): e2400002, 10.1002/ansa.202400002. 38948320 PMC11210747

[21] D. Silva , M. Thaitumu , G. Theodoridis , M. Witting , and H. Gika , “Volumetric Absorptive Microsampling in the Analysis of Endogenous Metabolites,” Metabolites 13, no. 10 (2023): 1038, 10.3390/metabo13101038. 37887363 PMC10609074

[22] P. Dodeja , S. Giannoutsos , S. Caritis , and R. Venkataramanan , “Applications of Volumetric Absorptive Microsampling Technique: A Systematic Critical Review,” Therapeutic Drug Monitoring 45, no. 4 (2023): 431–462, 10.1097/FTD.0000000000001083. 36917733

[23] M. Protti , R. Mandrioli , and M. L. Tutorial , “Volumetric Absorptive Microsampling (VAMS),” Analytica Chimica Acta 1046 (2019): 32–47, 10.1016/j.aca.2018.09.004. 30482302

[24] V. Londhe and M. Rajadhyaksha , “Opportunities and Obstacles for Microsampling Techniques in Bioanalysis: Special Focus on DBS and VAMS,” Journal of Pharmaceutical and Biomedical Analysis 182 (2020): 113102, 10.1016/j.jpba.2020.113102. 32014628

[25] J. D. Freeman , L. M. Rosman , J. D. Ratcliff , P. T. Strickland , D. R. Graham , and E. K. Silbergeld , “State of the Science in Dried Blood Spots,” Clinical Chemistry 64, no. 4 (2018): 656–679, 10.1373/clinchem.2017.275966. 29187355

[26] M. Luginbühl and S. Gaugler , “The Application of Fully Automated Dried Blood Spot Analysis for Liquid Chromatography‐Tandem Mass Spectrometry Using the CAMAG DBS‐MS 500 Autosampler,” Clinical Biochemistry 82 (2020): 33–39, 10.1016/j.clinbiochem.2020.02.007. 32087137

[27] H. Y. Tey and H. H. See , “A review of Recent Advances in Microsampling Techniques of Biological Fluids for Therapeutic Drug Monitoring,” Journal of Chromatography A 1635 (2021): 461731, 10.1016/j.chroma.2020.461731. 33285415

[28] P. Denniff and N. Spooner , “Volumetric Absorptive Microsampling: A Dried Sample Collection Technique for Quantitative Bioanalysis,” Analytical Chemistry 86, no. 16 (2014): 8489–8495, 10.1021/ac5022562. 25058158

[29] N. Youhnovski , L. Mayrand‐Provencher , E. R. Bérubé , et al., “Volumetric Absorptive Microsampling Combined With Impact‐Assisted Extraction for Hematocrit Effect Free Assays,” Bioanalysis 9, no. 22 (2017): 1761–1769, 10.4155/bio-2017-0167. 29148829

[30] S. Velghe and C. P. Stove , “Evaluation of the Capitainer‐B Microfluidic Device as a New Hematocrit‐Independent Alternative for Dried Blood Spot Collection,” Analytical Chemistry 90, no. 21 (2018): 12893–12899, 10.1021/acs.analchem.8b03512. 30256092

[31] O. Beck , M. Mellring , C. Löwbeer , S. Seferaj , and A. Helander , “Measurement of the Alcohol Biomarker Phosphatidylethanol (PEth) in Dried Blood Spots and Venous Blood‐Importance of Inhibition of Post‐Sampling Formation From Ethanol,” Analytical and Bioanalytical Chemistry 413, no. 22 (2021): 5601–5606, 10.1007/s00216-021-03211-z. 33590314 PMC8410693

[32] M. Menestrina Dewes , L. Cé da Silva , Y. Fazenda Meireles , et al., “Evaluation of the Tasso‐SST® Capillary Blood Microsampling Device for the Measurement of Endogenous Uracil Levels,” Clinical Biochemistry 107 (2022): 1–6, 10.1016/j.clinbiochem.2022.06.003. 35709975

[33] E. Wickremsinhe , A. Fantana , E. Berthier , et al., “Standard Venipuncture vs a Capillary Blood Collection Device for the Prospective Determination of Abnormal Liver Chemistry,” Journal of Applied Laboratory Medicine 8, no. 3 (2023): 535–550, 10.1093/jalm/jfac127. 36533519

[34] J. D. Jett , R. Beck , D. Tyutyunnyk , et al., “Validation of the Quantification of Phosphatidylethanol 16:0/18:1 Concentrations in TASSO‐M20 Devices,” Alcohol, Clinical & Experimental Research (Hoboken) 47, no. 4 (2023): 748–755, 10.1111/acer.15024. PMC10149590 36811188

[35] L. D. Noble , C. Dixon , A. Moran , et al., “Painless Capillary Blood Collection: A Rapid Evaluation of the Onflow Device,” Diagnostics (Basel) 13, no. 10 (2023): 1754, 10.3390/diagnostics13101754. 37238237 PMC10217062

[36] G. Lenk , S. Sandkvist , A. Pohanka , G. Stemme , O. Beck , and N. Roxhed , “A Disposable Sampling Device to Collect Volume‐Measured DBS Directly From a Fingerprick Onto DBS Paper,” Bioanalysis 7, no. 16 (2015): 2085–2094, 10.4155/bio.15.134. 26327187

[37] YourBio Health Revolutionizing Blood Collection, accessed November 15, 2024, https://yourbiohealth.com/.

[38] Loop Medical , For Fearless Blood Collection, accessed November 19, 2024, https://www.loop‐medical.com/.

[39] L. D. Roberts , A. L. Souza , R. E. Gerszten , and C. B. Clish , “Targeted Metabolomics,” in Current Protocols in Molecular Biology (John Wiley & Sons, 2012), 10.1002/0471142727.mb3002s98. PMC3334318 22470063

[40] O. Begou , H. G. Gika , I. D. Wilson , and G. Theodoridis , “Hyphenated MS‐Based Targeted Approaches in Metabolomics,” Analyst 142, no. 17 (2017): 3079–3100, 10.1039/c7an00812. 28792021

[41] S. Deprez , K. Van Uytfanghe , and C. P. Stove , “Liquid Chromatography‐Tandem Mass Spectrometry for Therapeutic Drug Monitoring of Immunosuppressants and Creatinine From a Single Dried Blood Spot Using the Capitainer® qDBS Device,” Analytica Chimica Acta 1242 (2023): 340797, 10.1016/j.aca.2023.340797. 36657891

[42] N. T. Vethe , A. Åsberg , A. M. Andersen , R. Heier Skauby , S. Bergan , and K. Midtvedt , “Clinical Performance of Volumetric Finger‐Prick Sampling for the Monitoring of Tacrolimus, Creatinine and Haemoglobin in Kidney Transplant Recipients,” British Journal of Clinical Pharmacology 89, no. 12 (2023): 3690–3701, 10.1111/bcp.15870. 37537150

[43] C. Torres , R. A. Muldrow , A. R. Naranjo , S. W. Cotten , C. C. Pierre , and D. N. Greene , “Development and Validation of an LC–MSMS Method to Quantify Creatinine From Dried Blood Spots,” Journal of Mass Spectrometry and Advances in the Clinical Lab 32 (2024): 50–59, 10.1016/j.jmsacl.2024.03.001. 38511102 PMC10950697

[44] R. S. Carling , Z. Barclay , N. Cantley , et al., “Investigation of the Relationship Between Phenylalanine in Venous Plasma and Capillary Blood Using Volumetric Blood Collection Devices,” JIMD Reports 64, no. 6 (2023): 468–476, 10.1002/jmd2.12398. 37927487 PMC10623100

[45] S. Catanese , C. F. Beuchel , T. Sawall , et al., “Biomarkers Related to Fatty Acid Oxidative Capacity Are Predictive for Continued Weight Loss in Cachectic Cancer Patients,” Journal of Cachexia, Sarcopenia and Muscle 12, no. 6 (2021): 2101–2110, 10.1002/jcsm.12817. 34636159 PMC8718041

[46] M. Boutin , P. Lavoie , M. Beaudon , et al., “Mass Spectrometry Analysis of Globotriaosylsphingosine and Its Analogues in Dried Blood Spots,” International Journal of Molecular Sciences 24, no. 4 (2023): 3223, 10.3390/ijms24043223. 36834643 PMC9966246

[47] M. J. Binks , A. S. Bleakley , G. Rathnayake , et al., “Can Dried Blood Spots be Used to Accurately Measure Vitamin D Metabolites?,” Clinica Chimica Acta 518 (2021): 70–77, 10.1016/j.cca.2021.03.003. 33713691

[48] H. B. Wang , X. Xiao , W. Dai , et al., “Rapid LC‐MS/MS Detection of 25‐Hydroxyvitamin D in Dried Blood Spots,” Analytica Chimica Acta 1283 (2023): 341964, 10.1016/j.aca.2023.341964. 37977788

[49] H. B. Wang , X. Xiao , X. Y. He , and S. T. Wang , “Advancing Towards Practice: A Novel LC‐MS/MS Method for Detecting Retinol in Dried Blood Spots,” Talanta 278 (2024): 126491, 10.1016/j.talanta.2024.126491. 38955103

[50] O. Salamin , T. Langer , J. Saugy , et al., “Comparison of Three Different Blood Matrices for the Quantification of Endogenous Steroids Using UHPLC‐MS/MS,” Drug Testing and Analysis 14, no. 11–12 (2022): 1920–1925, 10.1002/dta.3382. 36208447

[51] M. T. Ackermans , V. de Kleijne , F. Martens , and A. C. Heijboer , “Hematocrit and Standardization in DBS Analysis: A Practical Approach for Hormones Mainly Present in the Plasma Fraction,” Clinica Chimica Acta 520 (2021): 179–185, 10.1016/j.cca.2021.06.014. 34119531

[52] R. Matsuyama , T. Omata , M. Kageyama , R. Nakajima , M. Kanou , and K. Yamana , “Stabilization and Quantitative Measurement of Nicotinamide Adenine Dinucleotide in Human Whole Blood Using Dried Blood Spot Sampling,” Analytical and Bioanalytical Chemistry 415, no. 5 (2023): 775–785, 10.1007/s00216-022-04469-7. 36504284 PMC9741944

[53] A. Smith , K. Baumgartner , and C. Bositis , “Cirrhosis: Diagnosis and Management,” American Family Physician 100, no. 12 (2019): 759–770. 31845776

[54] J. W. Clinton , S. Kiparizoska , S. Aggarwal , S. Woo , W. Davis , and J. H. Lewis , “Drug‐Induced Liver Injury: Highlights and Controversies in the Recent Literature,” Drug Safety 44, no. 11 (2021): 1125–1149, 10.1007/s40264-021-01109-4. 34533782 PMC8447115

[55] A. D. Ricart , “Drug‐Induced Liver Injury in Oncology,” Annals of Oncology 28, no. 8 (2017): 2013–2020, 10.1093/annonc/mdx158. 28383671

[56] H. Akoglu , “User's Guide to Correlation Coefficients,” Turkish Journal of Emergency Medicine 18, no. 3 (2018): 91–93, 10.1016/j.tjem.2018.08.001. 30191186 PMC6107969

[57] G. B. McBride A Proposal for Strength‐of‐Agreement Criteria for Lin.s. Concordance Correlation Coefficient . NIWA Client Report: HAM2005‐062 (National Institute of Water & Atmospheric Research Ltd., 2005), https://www.medcalc.org/download/pdf/McBride2005.pdf.

[58] R. A. Koster , B. Greijdanus , J. W. Alffenaar , and D. J. Touw , “Dried Blood Spot Analysis of Creatinine With LC–MS/MS in Addition to Immunosuppressants Analysis,” Analytical and Bioanalytical Chemistry 407, no. 6 (2015): 1585–1594, 10.1007/s00216-014-8415-2. 25542583

[59] D. R. Koop , L. A. Bleyle , M. Munar , G. Cherala , and A. Al‐Uzri , “Analysis of Tacrolimus and Creatinine From a Single Dried Blood Spot Using Liquid Chromatography Tandem Mass Spectrometry,” Journal of Chromatography. B, Analytical Technologies in the Biomedical and Life Sciences 926 (2013): 54–61, 10.1016/j.jchromb.2013.02.035. 23548676 PMC4160148

[60] F. Bozzetti , “Forcing the Vicious Circle: Sarcopenia Increases Toxicity, Decreases Response to Chemotherapy and Worsens With Chemotherapy,” Annals of Oncology 28, no. 9 (2017): 2107–2118, 10.1093/annonc/mdx271. 28911059

[61] K. Fearon , F. Strasser , S. D. Anker , et al., “Definition and Classification of Cancer Cachexia: An International Consensus,” Lancet Oncology 12, no. 5 (2011): 489–495, 10.1016/S1470-2045(10)70218-7. 21296615

[62] R. Burkhardt , H. Kirsten , F. Beutner , et al., “Integration of Genome‐Wide SNP Data and Gene‐Expression Profiles Reveals Six Novel Loci and Regulatory Mechanisms for Amino Acids and Acylcarnitines in Whole Blood,” PLos Genetics 11, no. 9 (2015): e1005510, 10.1371/journal.pgen.1005510. 26401656 PMC4581711

[63] C. Muntean , I. M. Starcea , C. Stoica , and C. Banescu , “Clinical Characteristics, Renal Involvement, and Therapeutic Options of Pediatric Patients With Fabry Disease,” Frontiers in Pediatrics 10 (2022): 908657, 10.3389/fped.2022.908657. 35722479 PMC9198369

[64] L. Paim‐Marques , R. J. de Oliveira , and S. Appenzeller , “Multidisciplinary Management of Fabry Disease: Current Perspectives,” Journal of Multidisciplinary Healthcare 15 (2022): 485–495, 10.2147/JMDH.S290580. 35300178 PMC8922235

[65] D. Eyles , C. Anderson , P. Ko , et al., “A Sensitive LC/MS/MS Assay of 25OH Vitamin D3 and 25OH Vitamin D2 in Dried Blood Spots,” Clinica Chimica Acta 403, no. 1–2 (2009): 145–151, 10.1016/j.cca.2009.02.005. 19232332

[66] M. F. Holick , “The Vitamin D Deficiency Pandemic: Approaches for Diagnosis, Treatment and Prevention,” Reviews in Endocrine & Metabolic Disorders 18, no. 2 (2017): 153–165, 10.1007/s11154-017-9424-1. 28516265

[67] S. A. Tanumihardjo , R. M. Russell , C. B. Stephensen , et al., “Biomarkers of Nutrition for Development (BOND)‐Vitamin A Review,” Journal of Nutrition 146, no. 9 (2016): 1816S–1848S, 10.3945/jn.115.229708. 27511929 PMC4997277

[68] A. Carazo , K. Macáková , K. Matoušová , L. K. Krčmová , M. Protti , and P. Mladěnka , “Vitamin A Update: Forms, Sources, Kinetics, Detection, Function, Deficiency, Therapeutic Use and Toxicity,” Nutrients 13, no. 5 (2021): 1703, 10.3390/nu13051703. 34069881 PMC8157347

[69] M. Saugy and N. Leuenberger , “Antidoping: From Health Tests to the Athlete Biological Passport,” Drug Testing and Analysis 12, no. 5 (2020): 621–628, 10.1002/dta.2773. 31994337

[70] M. R. McReynolds , K. Chellappa , and J. A. Baur , “Age‐Related NAD^+^ Decline,” Experimental Gerontology 134 (2020): 110888, 10.1016/j.exger.2020.110888. 32097708 PMC7442590

[71] J. Clement , M. Wong , A. Poljak , P. Sachdev , and N. Braidy , “The Plasma NAD^+^ Metabolome Is Dysregulated in "Normal" Aging,” Rejuvenation Research 22, no. 2 (2019): 121–130, 10.1089/rej.2018.2077. 30124109 PMC6482912

[72] J. Yoshino , K. F. Mills , M. J. Yoon , and S. Imai , “Nicotinamide Mononucleotide, a Key NAD(+) Intermediate, Treats the Pathophysiology of Diet‐ and Age‐Induced Diabetes in Mice,” Cell Metabolism 14, no. 4 (2011): 528–536, 10.1016/j.cmet.2011.08.014. 21982712 PMC3204926

[73] S. A. Trammell , M. S. Schmidt , B. J. Weidemann , et al., “Nicotinamide Riboside is Uniquely and Orally Bioavailable in Mice and Humans,” Nature Communications 7 (2016): 12948, 10.1038/ncomms12948. PMC5062546 27721479

[74] A. Peluso , M. V. Damgaard , M. A. S. Mori , and J. T. Treebak , “Age‐Dependent Decline of NAD^+^‐Universal Truth or Confounded Consensus?,” Nutrients 14, no. 1 (2021): 101, 10.3390/nu14010101. 35010977 PMC8747183

[75] S. E. Airhart , L. M. Shireman , L. J. Risler , et al., “An Open‐Label, Non‐Randomized Study of the Pharmacokinetics of the Nutritional Supplement Nicotinamide Riboside (NR) and Its Effects on Blood NAD+ Levels in Healthy Volunteers,” PLoS ONE 12, no. 12 (2017): e0186459, 10.1371/journal.pone.0186459. 29211728 PMC5718430

[76] R. Chaleckis , I. Murakami , J. Takada , H. Kondoh , and M. Yanagida , “Individual Variability in Human Blood Metabolites Identifies Age‐Related Differences,” PNAS 113, no. 16 (2016): 4252–4259, 10.1073/pnas.1603023113. 27036001 PMC4843419

[77] P. I. Creeke , F. Dibari , E. Cheung , T. van den Briel , E. Kyroussis , and A. J. Seal , “Whole Blood NAD and NADP Concentrations Are Not Depressed in Subjects With Clinical Pellagra,” Journal of Nutrition 137, no. 9 (2007): 2013–2017, 10.1093/jn/137.9.2013. 17709435

[78] M. Tsalic , G. Bar‐Sela , A. Beny , B. Visel , and N. Haim , “Severe Toxicity Related to the 5‐Fluorouracil/Leucovorin Combination (The Mayo Clinic Regimen): A Prospective Study in Colorectal Cancer Patients,” American Journal of Clinical Oncology 26, no. 1 (2003): 103–106, 10.1097/01.COC.0000017526.55135.6D. 12576935

[79] C. Lunenburg , C. H. van der Wouden , M. Nijenhuis , et al., “Dutch Pharmacogenetics Working Group (DPWG) Guideline for the Gene‐Drug Interaction of DPYD and Fluoropyrimidines,” European Journal of Human Genetics 28, no. 4 (2020): 508–517, 10.1038/s41431-019-0540-0. 31745289 PMC7080718

[80] D. Meulendijks , L. M. Henricks , B. A. W. Jacobs , et al., “Pretreatment Serum Uracil Concentration as a Predictor of Severe and Fatal Fluoropyrimidine‐Associated Toxicity,” British Journal of Cancer 116, no. 11 (2017): 1415–1424, 10.1038/bjc.2017.94. 28427087 PMC5520099

[81] M. N. Fedoruk , “Virtual Drug Testing: Redefining Sample Collection in a Global Pandemic,” Bioanalysis 12, no. 11 (2020): 715–718, 10.4155/bio-2020-0119. 32530291 PMC7351082

[82] M. A. Javors , N. Hill‐Kapturczak , J. D. Roache , T. E. Karns‐Wright , and D. M. Dougherty , “Characterization of the Pharmacokinetics of Phosphatidylethanol 16:0/18:1 and 16:0/18:2 in Human Whole Blood After Alcohol Consumption in a Clinical Laboratory Study,” Alcoholism, Clinical and Experimental Research 40, no. 6 (2016): 1228–1234, 10.1111/acer.13062. 27130527 PMC4939838

[83] W. B. Dunn , D. Broadhurst , P. Begley , et al., “Human Serum Metabolome (HUSERMET) Consortium. Procedures for Large‐Scale Metabolic Profiling of Serum and Plasma Using Gas Chromatography and Liquid Chromatography Coupled to Mass Spectrometry,” Nature Protocols 6, no. 7 (2011): 1060–1083, 10.1038/nprot.2011.335. 21720319

[84] G. Theodoridis , H. Gika , D. Raftery , R. Goodacre , R. S. Plumb , and I. D. Wilson , “Ensuring Fact‐Based Metabolite Identification in Liquid Chromatography‐Mass Spectrometry‐Based Metabolomics,” Analytical Chemistry 95, no. 8 (2023): 3909–3916, 10.1021/acs.analchem.2c05192. 36791228 PMC9979140

[85] J. Drolet , V. Tolstikov , B. A. Williams , et al., “Integrated Metabolomics Assessment of Human Dried Blood Spots and Urine Strips,” Metabolites 7, no. 3 (2017): 35, 10.3390/metabo7030035. 28714878 PMC5618320

[86] C. Volani , C. Malfertheiner , G. Caprioli , et al., “VAMS‐Based Blood Capillary Sampling for Mass Spectrometry‐Based Human Metabolomics Studies,” Metabolites 13, no. 2 (2023): 146, 10.3390/metabo13020146. 36837765 PMC9958641

[87] M. P. Cala and R. J. Meesters , “Comparative Study on Microsampling Techniques in Metabolic Fingerprinting Studies Applying Gas Chromatography‐MS Analysis,” Bioanalysis 9, no. 17 (2017): 1329–1340, 10.4155/bio-2017-0037. 28901168

[88] N. H. Tobin , A. Murphy , F. Li , et al., “Comparison of Dried Blood Spot and Plasma Sampling for Untargeted Metabolomics,” Metabolomics 17, no. 7 (2021): 62, 10.1007/s11306-021-01813-3. 34164733 PMC8340475

[89] N. H. Tobin , F. Li , S. Brummel , et al., “Maternal HIV Infection and the Milk Microbiome,” Microbiome 12, no. 1 (2024): 182, 10.1186/s40168-024-01843-8. 39342403 PMC11439335

[90] H. H. Chiu , S. Y. Lin , C. G. Zhang , C. C. Tsai , S. C. Tang , and C. H. Kuo , “A Comparative Study of Plasma and Dried Blood Spot Metabolomics and Its Application to Diabetes Mellitus,” Clinica Chimica Acta 552 (2024): 117655, 10.1016/j.cca.2023.117655. 37977234

[91] X. Shen , R. Kellogg , D. J. Panyard , et al., “Multi‐Omics Microsampling for the Profiling of Lifestyle‐Associated Changes in Health,” Nature Biomedical Engineering 8, no. 1 (2024): 11–29, 10.1038/s41551-022-00999-8. PMC10805653 36658343

[92] M. N. Thaitumu , D. M. D. S. E. Silva , P. Louail , et al., “LC‐MS‐Based Global Metabolic Profiles of Alternative Blood Specimens Collected by Microsampling,” Metabolites 15, no. 1 (2025): 62, 10.3390/metabo15010062. 39852404 PMC11767270

[93] L. M. Petrick , M. M. Niedzwiecki , G. Dolios , et al., “Environmental influences on Child Health Outcomes. Effects of Storage Temperature and Time on Metabolite Profiles Measured in Dried Blood Spots, Dried Blood Microsamplers, and Plasma,” Science of the Total Environment 912 (2024): 169383, 10.1016/j.scitotenv.2023.169383. 38101622 PMC10842436

[94] Ó. Rolfsson , G. Paglia , M. Magnusdóttir , B. Ø. Palsson , and I. Thiele , “Inferring the Metabolism of Human Orphan Metabolites From Their Metabolic Network Context Affirms Human Gluconokinase Activity,” Biochemical Journal 449, no. 2 (2013): 427–435, 10.1042/BJ20120980. 23067238

[95] J. T. Yurkovich , D. C. Zielinski , L. Yang , et al., “Quantitative Time‐Course Metabolomics in Human Red Blood Cells Reveal the Temperature Dependence of Human Metabolic Networks,” Journal of Biological Chemistry 292, no. 48 (2017): 19556–19564, 10.1074/jbc.M117.804914. 29030425 PMC5712598

[96] G. Paglia , Ó. E. Sigurjónsson , A. Bordbar , et al., “Metabolic Fate of Adenine in Red Blood Cells During Storage in SAGM Solution,” Transfusion 56, no. 10 (2016): 2538–2547, 10.1111/trf.13740. 27491795

[97] M. Seip , R. Lindemann , P. Gjesdahl , and L. R. Gjessing , “Amino Acid Concentrations in Plasma and Erythrocytes in Aregeneratory and Haemolytic Anaemias,” Scandinavian Journal of Haematology 15, no. 3 (1975): 178–186, 10.1111/j.1600-0609.1975.tb01072.x. 1198060

[98] N. Rohatgi , T. K. Nielsen , S. P. Bjørn , et al., “Biochemical Characterization of Human Gluconokinase and the Proposed Metabolic Impact of Gluconic Acid as Determined by Constraint Based Metabolic Network Analysis,” PLoS ONE 9, no. 6 (2014): e98760, 10.1371/journal.pone.0098760. 24896608 PMC4045858

[99] S. Capiau , H. Veenhof , R. A. Koster , et al., “Official International Association for Therapeutic Drug Monitoring and Clinical Toxicology Guideline: Development and Validation of Dried Blood Spot‐Based Methods for Therapeutic Drug Monitoring,” Therapeutic Drug Monitoring 41, no. 4 (2019): 409–430, 10.1097/FTD.0000000000000643. 31268966

