# Supplementary material for: Connecting the Dots: Bridging Microsamples and Conventional Blood Matrices in Metabolic Biomarker Analysis
Source: Anal Sci Adv. 2025 Sep 14;6(2):e70044. doi: 10.1002/ansa.70044 (PMC12433758; doi:10.1002/ansa.70044)
Supplement: Supplementary file 1 — Supporting File 1: ansa70044‐sup‐0001‐SuppMat.docx [file ANSA-6-e70044-s001.docx]

**Supplementary**

**Table S1. Bµs and conventional matrices comparison in clinical chemistry panels by immunoassays**

| **Matrix/ Bµs device** | **Metabolites** | **Amount of sample** | **Regression and Bland Altman** | **Validation data** | **Reference** |
| --- | --- | --- | --- | --- | --- |
| TAP II, BDµM, and serum | BIL, BUN, cholesterol, CO2, creatinine, glucose, HDL, and TRIG | BIl, BUN, cholesterol, CO2, creatinine, glucose, TRIG - 2 µL. HDL 2.4 µL. | BIL, BUN, HDL, cholesterol, and TRIG R2 > 0.95, mean slope 1.1, mean bias 0.9 %, > 90% agreement except for TRIG.  Creatinine: R > 0.93, slope (0.868 to 0.987), mean bias (-8.1% to -10.9%). CO2 and glucose: R2 < 0.67. CO2 mean bias -14%.  Glucose mean bias was within CLIA despite < 55 % agreement. | NA | [2] |
| TASSO+SST and serum | Glucose, creatinine, phosphorous | Serum - 200 µL  TASSO+SST - NA | TS+SST (serum) vs serum: Deming regression intercept/slope - glucose 2.6133/1.0236, Creatinine -0.1059/1.1001, and phosphorous -0.2733/1.0788; linear regression CC/CCC glucose 0.9807/0.9744, creatinine 0.9928/0.9884, and phosphorus 0.9859/0.9832. TS+SST (LVB) vs serum: Deming regression intercept/slope - glucose -21.3489/0.9883, creatinine -0.03/1.0138, phosphorus -0.2039/1.0442; linear regression CC/CCC glucose 0.9399/0.8378, creatinine 0.9994/0.9989, phosphorous 1.0442/0.987. All regression parameters were within 95% CI. |  | [34] |
| OSG and serum | LDH and creatinine | NA | Creatinine: > 0.912 LDH: >0.673  Creatinine: Mean % bias -5.633 µmol/L, mean similarity 96%, similarity SD 2.7%, similarity CV 2.8%. LDH: Mean % bias 14.707 µmol/L, mean similarity 96%, similarity SD 104%, similarity CV 7%. | NA | [36] |

**Table S2. Bµs and conventional matrices comparison in analysis of metabolic markers of disease by mass spectrometry**

| **Matrix/ Bµs device** | **Metabolites** | **Amount of sample** | **Regression and Bland Altman** | **Validation data** | **Reference** |
| --- | --- | --- | --- | --- | --- |
| Venous Capitainer and plasma | Creatinine | Venous Capitainer - 10 μL.  Plasma - NA | NA | Accuracy < 6.9, Repeatability <4.5%, imprecision < 5.4% CV, Matric effects 101.9% to 85.7 at four different concentration levels. Method application: Bland Altman plot (n = 131): mean -0.8 μmol/L, SD +/- 1.96; Passing Bablok regression slope -2.501, intercept 1.014, rho 0.979. Passing Bablok regression of difference plot ((Venous Capitainer - plasma)/plasma against % HCT): slope -0.124 (within 95% CI), intercept 0.045, Pearson r -0.08. | [48] |
| Venous Capitainer, venous Mitra, and plasma | Creatinine | Venous Capitainer and venous Mitra - 10 µL.  Plasma - NA | Linear regression – Venous Capitainer vs Plasma : slope 0.75 and R2 0.994. VMitra vs Plasma : slope 0.91 and R2 0.91.  Venous Capitainer vs Plasma : -12.9% ± 13.2% Venous Mitra vs Plasma : 2.1% ± 13.5% | NA | [49] |
| Capillary DBS (supervised and unsupervised collection), venous DBS and plasma | Creatinine | Capillary DBS - 6 mm punch.  Plasma - NA |  | Total Precision : 5.2 % to 8.1 % Total Accuracy : 98.8 to 102.7 % Linearity Range : 0.3 to 20.0 mg/dL, R2 = 0.9964. % Difference VDBS vs plasma 2.6%. Matrix effects 105.4%, recovery 79.85%, and processing efficiency 103.07%. Average bias - 0.04 mg/dL (- 1.4%). 1) Supervised CDBS vs plasma: R2 0.9995, slope 0.954, and intercept 0.086. 2). Unsupervised CDBS vs plasma: R2 0.930, slope 1.3, and intercept 0.27. 3) VDBS vs plasma: R2 0.988, slope 0.945, and intercept 0.2. | [50] |
| Capillary Capitainer, capillary DBS, and plasma. | Phenylalanine | Capillary Capitainer - (2X10 µL). Capillary DBS - 2 drops. Plasma NA | Passing-Bablok regression: Capillary capitainer vs Plasma R2 0.992, slope -0.8909, intercept -3.67, Cusum test (p = 0.9889), RSD 45.5. All parameters were within 95% CI.  Capitainer vs Plasma : -13.3% 95% limits of agreement -29.03 to 2.44%. Capitainer vs CDBS : 9.42% | NA | [51] |
| Venous DBS and plasma | Amino acids and derivatives and carnitines. | Venous DBS – NA.  Plasma 50 µL. | Spearman correlation. Proline and alanine (rho ≥ 0.8); aminobutyric acid, phenylalanine, threonine, carnitine free, alanine/acetylcarnitine, decanoylcarnitine, glycine, citrulline (rho 0.6 to 0.79); leucine/isoleucine, tyrosine, sarcosine, methylhistidine, butyrylcarnitine, arginine, acetylcarnitine, arginine/citrulline, serine, glutarylcarnitine/lysine, hydroxyproline, pipecolic acid (rho 0.4 to 0.59).  According to spearman, rho correlation ≥ 0.8 is very strong, 0.6 to 0.79 is strong, 0.4 to 0.59 is moderate, 0.2 to 0.39 is low, and ＜0.2 the is no correlation. | NA | [52] |
| Capillary DBS, capillary Capitainer, venous DBS, venous Capitainer, and plasma. | Globotriaosylsphingosine (lyso-Gb3) & analogues | Capillary DBS - 50 µL, capillary Capitainer - 10 µL, venous DBS 50 µL, venous Capitainer - 10 µL, plasma - NA. | 1) Deming Regression for lyso-Gb3-13C6 and its analogues in CDBS vs Plasma: slope 0.825 to 0.993 and intercept -0.045 to 3.019. 2) Linear regression for lyso-Gb3-13C6 and its analogues in VDBS vs CDBS: slope 1.225 to 1.301 and intercept -2.576 to -0.170. 3) Linear regression for lyso-Gb3-13C6 and its analogues in VCapitainer vs Capillary capitainer: slope 0.979 to 1.178 and intercept -0.678 to 0.007. 3) Linear regression for lyso-Gb3-13C6 and its analogues in plasma vs DBS (venous and capillary) samples as a function of HCT: slope -0.070 to 8.107 and intercept -0.610 to 0.222. 4) Linear regression for lyso-Gb3-13C6 and its analogues in plasma vs Capitainer (venous and capillary) samples as function of HCT: slope -7.438 to 1.715 and intercept -0790 to 6.358. All parameters were within 95% CI.  Bland Altman for lyso-Gb3-13C6 and its analogues in DBS vs Plasma. Results given as mean +/- standard deviation.Lyso-Gb3 2.92 nmol/L +/-1.33 nmol/L  Lyso-Gb3 -28 Da 0.17 nmol/L +/- 0.15 nmol/L  Lyso-Gb3 -2 Da 0.19 nmol/L +/- 0.29 nmol/L  Lyso-Gb3 +16 Da 0.01 nmol/l +/- 0.1 nmol/L  Lyso-Gb3 +18 Da -0.25 nmol/L +/- 0.42 nmol/L  Lyso-Gb3 +34 Da -0.01 nmol/L +/- 0.21 nmol/L | Ranges for all lyso-Gb3-13C6 and its analogues 1) LOD : DBS : 0.10 - 0.32 nM on Capitainer : 0.15 - 0.40 nM Plasma : 0.06 - 0.29 nM 2) LOQ : DBS : 0.32 - 1.08 nM Capitainer : 0.55 - 1.34 nM plasma : 0.21 - 0.97 nM. Ranges for lyso-Gb3-13C6 1) Intra day Precision: DBS 3.17 - 4.67 % RSD, 2.94 - 5.55 4) Intra day accuracy: DBS 6.2 - 9.6 % bias, all 0.1 - 12.3 % bias. 2) Interday precision: DBS 2.6 - 5.2 % RSD 6) 2.1 - 6.9% RSD. 3) Interday accuracy: DBS 3.9 - 6.6 % bias, all Capitainer 0 - 5.1% bias. 4) Recovery: DBS extraction recovery DBS 66 - 71 % , Capitainer 66 - 68%; Solid phase extraction recovery DBS 71 - 74%, Capitainer 69 - 74 %. | [56] |
| Venous DBS, Capillary DBS, and plasma. | 25OHD3, 25OHD2, and 3-epi-25OHD3 | Venous DBS - 3.2 mm punch capillary DBS - 3.2 mm punch, and plasma - 10 µL. | 5% of the difference values were within two standard deviations (SD) of the mean. | Validated in DBS and plasma samples. 1) Precision < 11.5%. 2) Linear range: 25OHD3 (1 - 300 nmol/L), 25OHD2 (2 - 300 nmol/L), 3-epi-25OHD3 (1 -200 nmol/L). Linear regression plasma vs CDBS: 25OHD3 R^0.92, plasma vs VDBS R2 0.93, CDBS vs VDBS R2 0.97. | [59] |
| Venous DBS, capillary DBS, and plasma. | 25OHD3 and 25OHD2 | Venous DBS - 3.2 mm punch capillary DBS - 3.2 mm punch, and plasma - 20 µL. |  | Accuracy: 86.8 - 110.1%. Imprecision: 5.4 - 16.8%. Linear Range : 25OHD3 : 1-100 ng/mL, 25OHD2 : 1-100 ng/mL. LLOQ : 25OHD3 : 1 ng/mL, 25OHD2 : 1 ng/mL. Method comparison: 93% of metabolites were within +/- 20% difference. Passing-Bablok regression: 25OHD3 R2 0.966, slope 0.9820, intercept 0.2324, cusum test (P = 0.37). 25OHD2: 82% of samples met criteria. All parameters were within 95% CI. | [62] |
| Venous DBS, capillary DBS, and plasma. | Vitamin A | Venous DBS - 3.2 mm punch capillary DBS 10µL, and plasma - 20 µL. |  | Selectivity: No false positive signal with S/N > 3 was detected. Accuracy: < 10.6% bias. LLOQ: 70.7 ng/mL. Carryover-over effect: S/N < 3. Matrix effects: 95.7 - 112.9%. Accuracy: 89.7% - 102.8%. Precision < 9.8%. Linear Range : 70.7 - 1413.6 ng/mL. LLOQ: 70.7 ng/mL. Method comparison: Passing-Bablok regression slope 1.154, intercept -0.242, and cusum test (P = 1). All parameters were within 95% CI. Spearman rank correlation R2 0.888 (P < 0.0001). Bland-Altman mean 0.9804 (within 95% CI). 90% of samples were within +/- 20% bias criteria. | [63] |
| Venous DBS, serum, and plasma | TES, epitestosterone, A4, P, 17𝞪OHP, DHEA, DHT, corticosterone, cortisol, deoxycorticosterone, and 11-deoxycortisol | Venous DBS - 20 µL. Serum and plasma - 200 µL. | Passing-Bablok regression 1) VDBS vs serum: Testosterone slope -0.03, intercept 0.86, R2 0.98. 2) VDBS vs plasma: Testosterone slope -0.071, intercept 1.06, and R2 0.99. 3) VDBS vs serum androstenedione slope -0.12, intercept 1.04, R2 0.95 4) VDBS vs plasma androstenedione slope -0.04, intercept 1.29, R2 0.95. All parameters were within 95% CI.  Testosterone (T) : VDBS was 14% lower than serum, but aligned well with plasma Androsterone (A4) : VDBS was consistent with serum, but was overestimated by 34% in plasma. | NA | [67] |
| Venous DBS and plasma | 25OHD3 Testosterone | Venous DBS - 4 X 1/8 inch spots.  TES - 25 µL.  25OHD3 - 100 µL. | 25OHD3 1) HCT uncorrected VDBS vs plasma R2 0.971, slope 0.74, and intercept -2.20. 2) HCT corrected VDBS vs plasma R2 0.917, slope 1.24, and intercept -1.84. Testosterone 1) HCT uncorrected VDBS vs plasma R^ 0.994, slope 0.53, and intercept -0.09. 2) HCT corrected VDBS vs plasma R2 0.953, slope 0.89, and intercept -0.17. All parameters were within 95 CI. | Linear Range : DBS 25-OH vitamin D3 : 15-240 nmol/L Testosterone : 0.14-100 nmol/L Plasma 25-OH vitamin D3 : 4-340 nmol/L Testosterone : 0.1-100 nmol/L | [68] |
| Venous DBS and liquid blood. | NAD+ and NMN | 5 µL |  | LOQ : NAD+: 0.5 - 2 μM NMN: 0.25 - 2 μM | [75] |

**Table S3. Bµs and conventional matrices comparison in markers of toxicity and abuse**

| **Matrix/ Bµs device** | **Metabolites** | **Amount of sample** | **Regression and Bland Altman** | **Validation data** | **Reference** |
| --- | --- | --- | --- | --- | --- |
| TASSO+SST and plasma | Uracil | 50 µL | Passing-Bablok rank correlation coefficient (rs) 0.91, slope 0.9731, intercept -0.008627. All parameters were within 95% CI.  Limit of agreement -23.3% to 29.1%. | Linear Range 5-250 ng/mL; LLOQ 5 ng/mL; inter-assay precision 4.3% to 7.3%; intra-assay 3.4% to 6.1%; accuracy 98.8% to 102%; matrix effects average -6.42%; recovery 95% to 103.3%. | [33] |
| TASSO-M20, venous DBS, and liquid blood | PEth | 40 µL | 1) LVB vs TASSO-M20: R2 0.988 and slope 0.951 (n = 14). 2) For samples with PEth concentration range 0 to 200 ng/mL. LVB vs TASSO-M20 : R2 0.9438 and slope 0.8163 (n = 7).  TASSO-M20 vs LVB: Bias 4.95% and SD +/- 11.3%. PEth levels were 5% > in TASSO-M20 compared to LVB (n = 14). | NA | [35] |
| Venous DBS, venous Capitainer, venous Mitra, venous HDB, liquid venous blood | PEth | 10 µL |  |  | [32] |
